# Episignatures in practice: independent evaluation of published episignatures for the molecular diagnostics of ten neurodevelopmental disorders

**DOI:** 10.1038/s41431-023-01474-x

**Published:** 2023-10-23

**Authors:** Thomas Husson, François Lecoquierre, Gaël Nicolas, Anne-Claire Richard, Alexandra Afenjar, Séverine Audebert-Bellanger, Catherine Badens, Frédéric Bilan, Varoona Bizaoui, Anne Boland, Marie-Noëlle Bonnet-Dupeyron, Elise Brischoux-Boucher, Céline Bonnet, Marie Bournez, Odile Boute, Perrine Brunelle, Roseline Caumes, Perrine Charles, Nicolas Chassaing, Nicolas Chatron, Benjamin Cogné, Estelle Colin, Valérie Cormier-Daire, Rodolphe Dard, Benjamin Dauriat, Julian Delanne, Jean-François Deleuze, Florence Demurger, Anne-Sophie Denommé-Pichon, Christel Depienne, Anne Dieux, Christèle Dubourg, Patrick Edery, Salima El Chehadeh, Laurence Faivre, Patricia Fergelot, Mélanie Fradin, Aurore Garde, David Geneviève, Brigitte Gilbert-Dussardier, Cyril Goizet, Alice Goldenberg, Evan Gouy, Anne-Marie Guerrot, Anne Guimier, Inès Harzalla, Delphine Héron, Bertrand Isidor, Didier Lacombe, Xavier Le Guillou Horn, Boris Keren, Alma Kuechler, Elodie Lacaze, Alinoë Lavillaureix, Daphné Lehalle, Gaëtan Lesca, James Lespinasse, Jonathan Levy, Stanislas Lyonnet, Godeliève Morel, Nolwenn Jean-Marçais, Sandrine Marlin, Luisa Marsili, Cyril Mignot, Sophie Nambot, Mathilde Nizon, Robert Olaso, Laurent Pasquier, Laurine Perrin, Florence Petit, Veronique Pingault, Amélie Piton, Fabienne Prieur, Audrey Putoux, Marc Planes, Sylvie Odent, Chloé Quélin, Sylvia Quemener-Redon, Mélanie Rama, Marlène Rio, Massimiliano Rossi, Elise Schaefer, Sophie Rondeau, Pascale Saugier-Veber, Thomas Smol, Sabine Sigaudy, Renaud Touraine, Frederic Tran Mau-Them, Aurélien Trimouille, Julien Van Gils, Clémence Vanlerberghe, Valérie Vantalon, Gabriella Vera, Marie Vincent, Alban Ziegler, Olivier Guillin, Dominique Campion, Camille Charbonnier

**Affiliations:** 1 Univ Rouen Normandie, Inserm U1245 and CHU Rouen, Department of Psychiatry, F-76000 Rouen, France; 2 Department of Research, Centre hospitalier du Rouvray, Sotteville-Lès-Rouen, France; 3 Univ Rouen Normandie, Inserm U1245 and CHU Rouen, Department of Genetics and reference center for developmental disorders, F-76000 Rouen, France; 4 APHP. Sorbonne Université, Centre de Référence Malformations et maladies congénitales du cervelet et déficiences intellectuelles de causes rares, département de génétique et embryologie médicale, Hôpital Trousseau, F-75012 Paris, France; 5 Service de Génétique Médicale et Biologie de la Reproduction, CHU de Brest, Brest, France; 6 Aix Marseille Univ, INSERM, MMG, Marseille, France; APHM, service de génétique, Marseille, France; 7 CHU de Poitiers, Service de Génétique Médicale and Université de Poitiers, INSERM U1084, LNEC, F- 86000 Poitiers, France; 8 Service de génétique et neurodéveloppement, Pôle de Santé Mentale Enfant et Adolescent, Centre Hospitalier de l’Estran, Pontorson, France; 9 Université Paris-Saclay, CEA, Centre National de Recherche en Génomique Humaine (CNRGH), 91057 Evry, France; 10 Consultations de Génétique, Centre Hospitalier de Valence, 26953 Valence, France; 11 Centre de génétique humaine, CHU Besancon, Universite de Bourgogne Franche-Comte, Besancon, France; 12 Laboratoire de génétique médicale, CHRU Nancy, Nancy, France; 13 Université de Lorraine, INSERM UMR_S1256, NGERE, F-54000 Nancy, France; 14 Centre de Génétique et Centre de Référence Anomalies du Développement et Syndromes Malformatifs, FHU TRANSLAD, Hôpital d’Enfants, CHU Dijon, Dijon, France; 15 CHU Lille, Clinique de génétique Guy Fontaine, F-59000 Lille, France; 16 Univ. Lille, CHU Lille, ULR 7364 - RADEME - Institut de Génétique Médicale, F-59000 Lille, France; 17 Département de génétique clinique, centre de référence des déficiences intellectuelles de causes rares, GHU Pitié Salpêtrière, Paris, France; 18 Service de Génétique Médicale, CHU Toulouse, Toulouse, France; 19 Service de Génétique, Hospices Civils de Lyon, Lyon, France; 20 Institute NeuroMyoGène, Laboratoire Physiopathologie et Génétique du Neurone et du Muscle, CNRS UMR 5261 -INSERM U1315, Université de Lyon - Université Claude Bernard Lyon 1, Lyon, France; 21 Nantes Université, CNRS, INSERM, l’institut du thorax, F-44000 Nantes, France; 22 CHU Nantes, Service de Génétique Médicale, Nantes Université, CNRS, INSERM, l’institut du thorax, F-44000 Nantes, France; 23 Service de Génétique Médicale, CHU Angers, Angers, France; 24 Service de médecine génomique des maladies rares, hôpital Necker Enfants Malades, Paris, France; 25 Université Paris Cité, INSERM UMR 1163, Institut Imagine, Paris, France; 26 Génétique médicale, CHI Poissy-Saint-Germain-en-Laye, 78300 Poissy, France; 27 Service de cytogénétique et génétique médicale, Hôpital Mère Enfant, CHU Limoges, Limoges, France; 28 Centre de Génétique et Centre de référence « Déficiences intellectuelles de causes rares », FHU TRANSLAD, Hôpital d’Enfants, CHU Dijon, Dijon, France; 29 Équipe GAD, INSERM UMR1231, Université de Bourgogne, Dijon, France; 30 Service de génétique, CHBA, Vannes, France; 31 Unité Fonctionnelle Innovation en Diagnostic génomique des maladies rares, FHU-TRANSLAD, CHU Dijon, Bourgogne, Dijon, France; 32 Institute of Human Genetics, University Hospital Essen, University Duisburg-Essen, Essen, Germany; 33 Service de Génétique Moléculaire et Génomique, CHU Pontchaillou, Rennes, France; 34 Université de Rennes, IGDR (Institut de Génétique et Développement), CNRS UMR 6290, INSERM ERL 1305, Rennes, France; 35 Université Claude Bernard Lyon 1, INSERM, CNRS, Centre de Recherche en Neurosciences de Lyon CRNL U1028 UMR5292, Genetics of Neurodevelopment (GENDEV) Team, 69500 Bron, France; 36 Service de Génétique Médicale, Institut de Génétique Médicale d’Alsace (IGMA), Hôpitaux Universitaires de Strasbourg, Strasbourg, France; 37 Institut de Génétique et de Biologie Moléculaire et Cellulaire (IGBMC), INSERM U1258, CNRS-UMR7104, Université de Strasbourg, Illkirch-Graffenstaden, France; 38 Laboratoire de Génétique Médicale, UMRS 1112, Institut de Génétique Médicale d’Alsace (IGMA), Université de Strasbourg et INSERM, Strasbourg, France; 39 Department of Medical Genetics, University Hospital of Bordeaux and INSERM U1211, University of Bordeaux, Bordeaux, France; 40 Service de Génétique Clinique, Centre de Référence Anomalies du Développement de l’Ouest, CHU Rennes, Rennes, France; 41 Université Montpellier, Inserm U1183, Montpellier, France; 42 Centre de référence anomalies du développement et syndromes malformatifs, Génétique Clinique, CHU Montpellier, Montpellier, France; 43 CHU de Poitiers, Service de Génétique Médicale, F-86000 Poitiers, France; 44 NRGEN team, Univ. Bordeaux, CNRS, INCIA, UMR 5287, EPHE, F-33000 Bordeaux, France; 45 Centre de Référence Maladies Rares Neurogénétique, Service de Génétique Médicale, Bordeaux University Hospital (CHU Bordeaux), Bordeaux, France; 46 Génétique et neurobiologie de C.elegans, MéLis (CNRS UMR 5284 -INSERM U1314), Institut NeuroMyogene, Université Claude Bernard Lyon 1, Lyon, France; 47 Service de médecine génomique des maladies rares - GHU Necker- Enfants malades, Paris, France; 48 Service de Génétique, CHU Hôpital Nord, Saint Etienne, France; 49 APHP.Sorbonne Université, Département de Génétique, Hôpital Trousseau & Groupe Hospitalier Pitié-Salpêtrière, Paris, France; 50 CHU de Poitiers, Service de Génétique Médicale, F – 86000 Poitiers, France; 51 Université de Poitiers, CNRS 7348, LabCom I3M-Dactim mis / LMA, F-86000 Poitiers, France; 52 Département de génétique médicale, Hôpital Pitié-Salpêtrière, AP-HP.Sorbonne Université, 75013 Paris, France; 53 Le Havre Hospital, Department of Medical Genetics, F 76600 Le Havre, France; 54 CHU Rennes, Service de Génétique Clinique, Centre de Référence Anomalies du développement, FHU GenOMedS, Univ Rennes, CNRS, INSERM, IGDR, UMR 6290, ERL U1305 Rennes, France; 55 UF de génétique médicale, Centre Hospitalier Métropole Savoie, BP 31135, 73011 Chambéry, France; 56 Genetics Department, AP-HP, Robert-Debré University Hospital, Paris, France; 57 Service de médecine génomique des maladies rares, Hôpital Universitaire Necker-Enfants malades, APHP, Paris, France; 58 Laboratoire embryologie et génétique des malformations, Institut Imagine, UMR-II63, INSERM, Université Paris Cité, GHU Necker- Enfants malades, Paris, France; 59 CHU Rennes, Service de Génétique Clinique, Centre de Référence Anomalies du développement, FHU GenOMedS, Rennes, France; 60 Médecine Physique et Réadaptation pédiatrique CHU Saint-Etienne, 42055 Saint-Etienne Cedex 2, France; 61 Service de Médecine Génomique des maladies rares, AP-HP. Centre, Hôpital Necker-Enfants Malades, F-75015 Paris, France; 62 Université Paris Cité, Institut Imagine, Inserm U1163, F-75015 Paris, France; 63 Laboratoire de diagnostic génétique, IGMA, Hôpitaux Universitaires de Strasbourg, Strasbourg, France; 64 Univ Brest, Inserm, EFS, UMR 1078, GGB, F-29200 Brest, France; 65 Centre de Référence Déficiences Intellectuelles de causes rares, Brest, France; 66 CHU Lille - Institut de Génétique Médicale, F-59000 Lille, France; 67 Service de Génétique Médicale -Institut de Génétique Médicale d’Alsace - CHU Strasbourg, Strasbourg, France; 68 Aix Marseille Univ, INSERM, MMG, CRMR syndromes malformatifs et anomalies du développement, département de génétique, APHM Hopital Timone, Marseille, France; 69 Service de Pathologie, CHU Bordeaux, Bordeaux, France; 70 Inserm U1211 MRGM, Université de Bordeaux, Bordeaux, France; 71 Centre d’Excellence InovAND-Service de psychiatrie de l’enfant et de l’adolescent-CHU Robert Debré, Paris, France; 72 Univ Rouen Normandie, Inserm U1245 and CHU Rouen, Department of Biostatistics, F-76000 Rouen, France

**Keywords:** Diagnostic markers, Genetics research, Neurodevelopmental disorders, DNA methylation

## Abstract

Variants of uncertain significance (VUS) are a significant issue for the molecular diagnosis of rare diseases. The publication of episignatures as effective biomarkers of certain Mendelian neurodevelopmental disorders has raised hopes to help classify VUS. However, prediction abilities of most published episignatures have not been independently investigated yet, which is a prerequisite for an informed and rigorous use in a diagnostic setting. We generated DNA methylation data from 101 carriers of (likely) pathogenic variants in ten different genes, 57 VUS carriers, and 25 healthy controls. Combining published episignature information and new validation data with a k-nearest-neighbour classifier within a leave-one-out scheme, we provide unbiased specificity and sensitivity estimates for each of the signatures. Our procedure reached 100% specificity, but the sensitivities unexpectedly spanned a very large spectrum. While *ATRX, DNMT3A, KMT2D*, and *NSD1* signatures displayed a 100% sensitivity, *CREBBP-RSTS* and one of the *CHD8* signatures reached <40% sensitivity on our dataset. Remaining Cornelia de Lange syndrome, *KMT2A*, *KDM5C* and *CHD7* signatures reached 70–100% sensitivity at best with unstable performances, suffering from heterogeneous methylation profiles among cases and rare discordant samples. Our results call for cautiousness and demonstrate that episignatures do not perform equally well. Some signatures are ready for confident use in a diagnostic setting. Yet, it is imperative to characterise the actual validity perimeter and interpretation of each episignature with the help of larger validation sample sizes and in a broader set of episignatures.

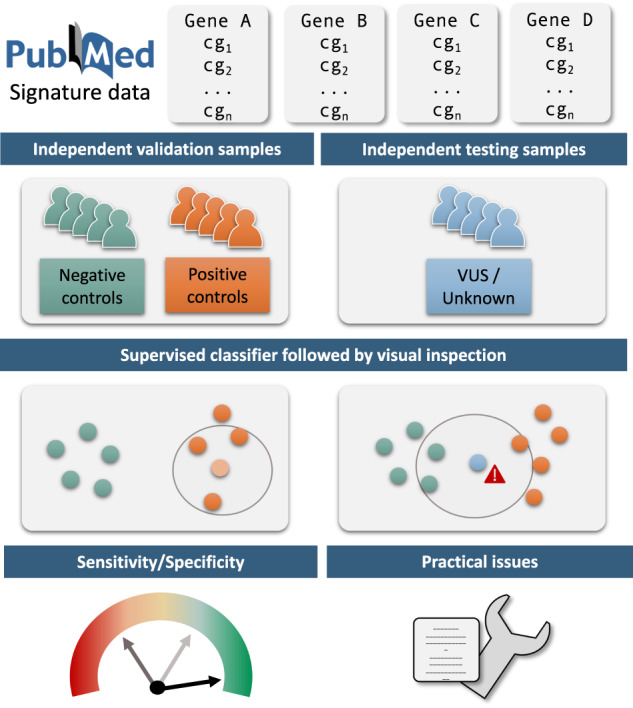

## Introduction

The efficient etiological diagnosis of neurodevelopmental disorders (NDDs) represents an important matter of public health. However, behind a single denomination, NDDs encompass a wide spectrum of clinical manifestations, arising from a large and heterogeneous set of rare disorders, from monogenic, Mendelian disorders to non-syndromic presentations with variable expression of traits. Providing a timely and accurate molecular diagnosis of monogenic disorders is of utmost importance to patients and their families. A precise and definite molecular diagnosis helps define personalised care for each patient, paves the way to genetic counselling and offers the possibility of prenatal diagnosis. In some cases, the final diagnosis is tediously obtained after years of diagnostic odyssey. Some others remain unsolved.

Large trio-based exome and genome sequencing studies have shown that, beyond environmental factors, genetic factors considerably contribute to the determinism of NDDs. However, genetic causes are extremely heterogeneous. Thousands of genes are now considered to contribute to the genetic etiology of NDDs. In recent years, implementation of exome or genome sequencing in a diagnostic setting has largely contributed to an increase in the rate of patients with definite diagnosis [1]. An important source of improvement is that diagnostic yields result from the rigorous interpretation of (likely) pathogenic (class 4–5) variants according to the ACMG-AMP (American College of Medical Genetics and Genomics—American for Molecular Pathology)—classification [2]. However, the interpretation process is plagued by a large number of variants of uncertain significance (VUS, ACMG-AMP class-3 variants). Finding informative clues to classify VUS as benign or pathogenic is one of the most important tasks in the post-sequencing era of medical genomics.

In the last decade, a new and promising strategy has emerged as an efficient alternative to hard-to-design and time-consuming functional studies to realise this task. The rationale is that a large number of NDD genes are involved in the regulation of gene expression, from transcription factors/regulators to critical players of the dynamic 3D-chromatin organisation and the epigenetic machinery, including histone and DNA methylation regulation [3, 4]. Therefore, the idea is to investigate the pathogenicity of genetic variants through the identification of episignatures, namely vast disruptions in DNA methylation patterns, that are characteristic of affected samples. Over the last 10 years, several research groups have published such episignatures, using Illumina 450 K and more recently 850 K EPIC Infimium Beadchips [5–21]. In practice, episignatures most often result from a two-step approach. First, a genome-wide analysis identifies a set of CpG positions that are differentially methylated between patients affected by a given condition due to a pathogenic variant in a specific set of genes, unique gene or even a specific functional domain, and unaffected controls. Then, a small subset of these positions are combined within a supervised classifier as the final episignature. Support Vector Machines (SVM) are the most common machine-learning approach adopted at this stage in the literature, but any supervised classifier allowing for high-dimensional datasets is possible. Resulting predictions can be used in combination with other arguments from the ACMG-AMP variants interpretation recommendations to discriminate whether a VUS is eventually pathogenic or not.

Several research groups have repeatedly provided evidence of the effectiveness of this concept from a general point of view [18, 22–25]. More than 50 episignatures have been reported in the literature so far, in the context of more than 60 syndromes [11, 20]. However, for most episignatures, the robustness, reproducibility, and actual sensitivity still need to be assessed on independent datasets, for the following reasons. Firstly, DNA methylation datasets are a perfect example of what statisticians call “ultra-high-dimensional datasets” because the number of CpG positions, here 450 K/800 K, is much larger than the sample size, with sometimes no more than 5 patients [11]. Combining this high-dimensional curse with technical or biological biases, episignatures are prone to overfitting, namely that both the selection step and predictive model might over adjust to the specificities and randomness of the discovery set but will not generalise well to other datasets, with new individuals, generated with different platforms or pipelines. As a result, there is a crucial need to validate the reproducibility of episignature position sets on independent datasets, before we can generalise their use in diagnostic setting. Secondly, because the overall methodology is still varying across the literature [26], an independent evaluation of episignature diagnostic performances (sensitivity and specificity) requires a single neutral and unified framework in which all signatures would be put on the same level for comparison.

With a focus on ten neurodevelopmental disorders, our objective was two-fold. Primarily, we aimed to validate the ability of the 16 corresponding episignatures in the literature to discriminate cases from controls on an independent validation dataset. This ability was quantified through an unbiased assessment of the diagnostic accuracy of corresponding episignatures, in terms of specificity, inter-syndrome specificity and sensitivity. Secondly, we applied our prediction strategy to the classification of VUS and describe the practical challenges encountered. For these purposes, we generated an independent validation and testing set of a target of ten new carrier samples of each tested episignature along with aged and sex-matched controls as well as VUS carriers. Because the actual classification algorithms as well as most of the raw data that would be required to replicate the training steps not always openly-shared, we obtained accurate sensitivity and specificity estimates from our validation dataset by embedding a multiple class k-Nearest Neighbour classification algorithm (kNN) into a leave-one out scheme to estimate the predictive abilities of each published list of probes and then apply it without the leave-one out strategy to VUS samples.

## Methods

### Sample collection

We leveraged a nation-wide collaborative effort to gather DNA samples isolated from fresh blood of probands harbouring likely pathogenic or pathogenic (LP/P) variants in a list of twelve genes spanning ten neuro-developmental disorders: *ATRX* (Alpha-thalassemia/mental retardation syndrome, MIM#301040)*, CHD7* (CHARGE syndrome, MIM#214800)*, CHD8* (Autism, Susceptibility to, 18 (AUTS18), MIM#615032)*, CREBBP (*Rubinstein-Taybi syndrome 1 (RSTS), MIM#180849), *DNMT3A* (Tatton-Brown-Rahman syndrome (*TBRS)*, MIM#615879)*, KDM5C* (Intellectual developmental disorder, X-linked syndromic, Claes-Jensen type (MRXSCJ), MIM#300534)*, KMT2D* (Kabuki syndrome 1 MIM# 147920)*, KMT2A* (Wiedemann-Steiner syndrome, MIM#605130)*, NIPBL/SMC1A/SMC3* (Cornelia de Lange syndrome 1–3 (CdL), MIM#122470,MIM#300590, and MIM#610759), *NSD1* (Sotos syndrome, MIM# 117550), as described in Table 1. All these variants were classified as class 4 or 5 variants according to ACMG-AMP interpretation guidelines by experienced geneticists from a network of reference centers for developmental abnormalities in France. Only *CREBBP* variants associated with RSTS were included in this dataset, namely missense variants within the first 29 exons and protein-truncating variants. We included germline variations detectable from DNA extracted from whole blood. Along with 25 normal controls free of any neurodevelopmental condition, these samples whose status is perfectly and a priori known constitute the *validation set* and are described in Supplementary Table 1.

**Table 1:** Description of episignatures under investigation.

Episignature name	*n* probes (% after QC)	*n* samples discovery/testing	% probes with Δ*β* > 0.10	% probes with Δ*β* > 0.05	Source
*ATRX*	101 (100%)	13/5	70%	97%	Aref-Eshghi, E. et al. Evaluation of DNA Methylation Episignatures for Diagnosis and Phenotype Correlations in 42 Mendelian Neurodevelopmental Disorders. Am J Hum Genet 106, 356–370 (2020).
AUTS18/*CHD8*_1	491 (79.6%)	7/13	4%	22%	Siu, M. T. et al. Functional DNA methylation signatures for autism spectrum disorder genomic risk loci: 16p11.2 deletions and CHD8 variants. Clin Epigenetics 11, 103 (2019).
AUTS18/*CHD8*_2	103 (99%)	5/0	5%	35%	Aref-Eshghi, E. et al. Evaluation of DNA Methylation Episignatures for Diagnosis and Phenotype Correlations in 42 Mendelian Neurodevelopmental Disorders. Am J Hum Genet 106, 356–370 (2020).
CdL	128 (99.2%)	31/10	11%	39%	Aref-Eshghi, E. et al. Evaluation of DNA Methylation Episignatures for Diagnosis and Phenotype Correlations in 42 Mendelian Neurodevelopmental Disorders. Am J Hum Genet 106, 356–370 (2020).
*CHARGE/CHD7_1*	165 (93.3%)	19/20	21%	50%	Butcher, D. T. et al. CHARGE and Kabuki Syndromes: Gene-Specific DNA Methylation Signatures Identify Epigenetic Mechanisms Linking These Clinically Overlapping Conditions. The American Journal of Human Genetics 100, 773–788 (2017).
CHARGE/*CHD7*_2	148 (100%)	45/15	20%	69%	Aref-Eshghi, E. et al. Evaluation of DNA Methylation Episignatures for Diagnosis and Phenotype Correlations in 42 Mendelian Neurodevelopmental Disorders. Am J Hum Genet 106, 356–370 (2020).
Kabuki/*KMT2D_1*	221 (92.8%)	11/8	39%	86%	Butcher, D. T. et al. CHARGE and Kabuki Syndromes: Gene-Specific DNA Methylation Signatures Identify Epigenetic Mechanisms Linking These Clinically Overlapping Conditions. The American Journal of Human Genetics 100, 773–788 (2017).
Kabuki/KMT2D_2	153 (100%)	66/21	35%	88%	Aref-Eshghi, E. et al. Evaluation of DNA Methylation Episignatures for Diagnosis and Phenotype Correlations in 42 Mendelian Neurodevelopmental Disorders. Am J Hum Genet 106, 356–370 (2020).
MRXSCJ/*KDM5C*_1	53 (96.2%)	10/0	27%	61%	Grafodatskaya, D. et al. Multilocus loss of DNA methylation in individuals with mutations in the histone H3 Lysine 4 Demethylase KDM5C. BMC Medical Genomics 6, 1 (2013).
MRXSCJ/*KDM5C*_2	127 (100%)	26/8	34%	72%	Aref-Eshghi, E. et al. Evaluation of DNA Methylation Episignatures for Diagnosis and Phenotype Correlations in 42 Mendelian Neurodevelopmental Disorders. Am J Hum Genet 106, 356–370 (2020).
RSTS/*CREBBP*	139 (100%)	30/9	0%	14%	Aref-Eshghi, E. et al. Evaluation of DNA Methylation Episignatures for Diagnosis and Phenotype Correlations in 42 Mendelian Neurodevelopmental Disorders. Am J Hum Genet 106, 356–370 (2020).
Sotos/*NSD1*_1	7085 (92.8%)	19/19	97%	100%	Choufani, S. et al. NSD1 mutations generate a genome-wide DNA methylation signature. Nat Commun 6, 10207 (2015).
Sotos/*NSD1*_2	112 (99.1%)	47/15	100%	100%	Aref-Eshghi, E. et al. Evaluation of DNA Methylation Episignatures for Diagnosis and Phenotype Correlations in 42 Mendelian Neurodevelopmental Disorders. Am J Hum Genet 106, 356–370 (2020).
TBRS/*DNMT3A*	139 (99.3%)	10/4	91%	99%	Aref-Eshghi, E. et al. Evaluation of DNA Methylation Episignatures for Diagnosis and Phenotype Correlations in 42 Mendelian Neurodevelopmental Disorders. Am J Hum Genet 106, 356–370 (2020).
WDSTS/*KMT2A*_1	104 (100%)	12/4	38%	76%	Aref-Eshghi, E. et al. Evaluation of DNA Methylation Episignatures for Diagnosis and Phenotype Correlations in 42 Mendelian Neurodevelopmental Disorders. Am J Hum Genet 106, 356–370 (2020).
WDSTS/*KMT2A*_2	207 (100%)	41/?	39%	93%	Foroutan, A. et al. Clinical Utility of a Unique Genome-Wide DNA Methylation Signature for KMT2A-Related Syndrome. Int J Mol Sci 23, 1815 (2022).

As a proof of concept, we also constituted a *testing set*, including 57 VUS carriers, as well as 8 “clinical hypothesis” cases, namely probands without definite molecular diagnostic despite a suggestive clinical presentation of a syndrome within one of the ten syndromes under investigation, as described in Supplementary Table 1.

All patients or legal representatives provided informed written consent for exome/genome analyses in a medical setting that contains a query on the use of residual samples for research. This genetic study was approved by our legal ethics committee.

### DNA methylation analysis

Genomic DNA was extracted from whole blood and bisulfite converted. DNA methylation profile was then derived using Illumina’s Infinium EPIC array v1.0, in accordance with the manufacturer’s protocol. Carriers were randomly assigned a chip position while controls were homogeneously distributed over all rows of the chips. DNA methylation arrays were generated either by Diagenode SA, Liège, Belgium (*n* = 174) or by the Centre National de Recherche en Génomique Humaine (CNRGH), Evry, France (*n* = 22). Except for CNRGH samples which only contained AUTS18 cases and 3 CdL VUS carriers, unaffected controls and patients were evenly assigned over the beadchips and eight beadchip cells. These data were newly generated without overlap with original episignature training sets.

Raw IDAT data were processed and normalised using the Meffil R package [27]. This package efficiently handles large DNA methylation datasets. Briefly, probes which failed methylation detection (detection *p* value > 0.01) in more than 5% of samples were removed. Samples with >1% of failed probes or an outlier methylation distribution (methylation/unmethylation ≥ 3 s.d. from mean) were flagged. Three samples from the testing set (MRXSCJ_17, Sotos_12 and Sotos_18) failed these quality controls and were excluded from further steps. Remaining samples were functionally normalised together as advocated in the Meffil documentation, with adjustment on array, sentrix column and row, before computing β-values.

Several predictions derived from methylation values were added to the sample information table. Sex predictions were extracted from the standard meffil normalised object. No inconsistencies between reported and predicted sex were noted. Blood cell counts were estimated with the meffil.cell.count.estimates function. DNA methylation age was predicted with the DNAmAge function from the methyclock R package [28]. Among all available epigenetic clocks, the skinHorvath clock [29], which was trained on skin and blood 450 K samples, displayed the strongest correlation with actual age at inclusion on our dataset (Pearson correlation *r* = 0.91, 95% confidence interval [0.88–0.93]), and was thereby used as age predictor. For statistical analyses, we adjusted on age and sex predictions for consistency across samples with known or unknown age and sex.

### Statistical analysis

We performed the following steps for each episignature, separately.

#### Literature data extraction

Episignature probes were not selected from our data. Instead, the probe list was retrieved from supplemental information of the corresponding publication (Table 1). All analyses regarding this episignature were then restricted to this specific list of probes. All probes are listed in Supplementary Table 3.

#### Case-control gap

To assess whether the episignature strength was reproduced on our dataset, we computed the proportion of CpG positions whose absolute average difference between cases and controls met the 5 and 10% thresholds that are typically required at discovery.

#### Adjustment for confounders

A linear regression model was fitted to adjust probe β-values on predicted age, sex and cell composition, which are all well-known confounders of methylation levels. Unless otherwise stated, residuals from this model were used in the following steps. Average residual methylation levels among pathogenic variant carriers and controls are given in Supplementary Table 3.

#### Evaluation of predictive abilities

Following a leave-one-out scheme, the kNN implementation from the “class” R package was used to predict the status (case or control) of every validation sample, case or control, using all remaining validation samples as training set. To guarantee high inter-syndrome specificity, a multiclass kNN was fitted to the full validation set simultaneously. The process was repeated for each validation sample. True positives and true negatives were then summarised into sensitivity and specificity estimations along with 95% confidence intervals based on an exact binomial distribution. To challenge the robustness of our results, we let the number k of nearest neighbours and the required level of consensus vary. Parameters ranged from “2/2”, perfect consensus between the two nearest neighbours, to “5/5”, perfect consensus between the five nearest neighbours. For 4 and 5 nearest neighbour predictions, because some syndromes display close signature profiles, we also allowed for the possibility of one discordant nearest neighbour (respectively “3/4” and “4/5”). *KDM5C* and *ATRX* genes being located on the X chromosome, it is expected that female carriers should not fully present the same episignature as male carriers. We therefore restricted sensitivity computations for these two genes to male samples. Inter-syndrome specificity was computed from other variant carriers.

Three subsidiary analyses were run to gain perspective:

- Four original episignatures were accessible through the EpigenCentral (https://epigen.ccm.sickkids.ca/) open-access web-portal [25, 30]. We loaded our dataset onto the platform and followed user guide recommended practices.
- To evaluate the gap between kNN and SVM predictors, an SVM algorithm (using the e1071 package default parameters) was fitted to residuals.
- To evaluate the impact of age, sex and blood composition adjustment, a kNN was fitted on normalised betas without adjustment for age, sex and blood composition.

#### Visual representation

The reliability of case/control discrimination was visually inspected on the first two principal components as well as on a heatmap of DNA methylation residuals of episignature probes.

#### VUS classification

Finally, VUS and clinical hypothesis samples from the testing set were classified using the “3/4” kNN algorithm.

## Results

### Recruitment

A total of 101 samples from ten neuro-developmental disorders described in Table 1 along with 25 age and sex-matched controls free from neurodevelopmental disorders (validation set), 57 VUS carriers and 8 clinical hypothesis samples (testing set) passed meffil quality checks and were included in our evaluation.

### Independent evaluation of episignature prediction accuracy

The reproducibility of episignatures was first assessed by computing the proportion of probes exceeding a 5 or 10% absolute average difference between cases and controls, as displayed in Table 1. Except *ATRX, DNMT3A* and both *NSD1* signatures, all signatures displayed less than 50% positions above the 10% threshold. CdL, CHD8_2, CHD8_1 and CREBBP showed the lowest reproducibility, with <40% original positions reaching the 5% threshold, namely 39%, 35%, 22% and 14% respectively.

For all episignatures, the main available information is the set of probes used for prediction. Average β or Δβ profiles are often provided but they find themselves contaminated by batch effects. The prediction algorithm itself is never shared. In our efforts to validate most faithfully the performances of each episignature, we therefore combined signature probe information with our independent validation and testing datasets which could be handled robustly from raw datafiles. Namely, because methylation levels strongly depend on age, sex and cellular composition, all analyses and graphical representations were made from residual methylation levels, after adjustment for such major confounders. For every episignature, we combined a quantitative evaluation of prediction performances as obtained from kNN clustering with a visual inspection of DNA methylation profiles on principal components and heatmap. Sensitivity and specificity were estimated using a leave-one-out strategy on our independent dataset, guaranteeing the absence of overfitting and thereby unbiased estimates.

**Fig. 1:**
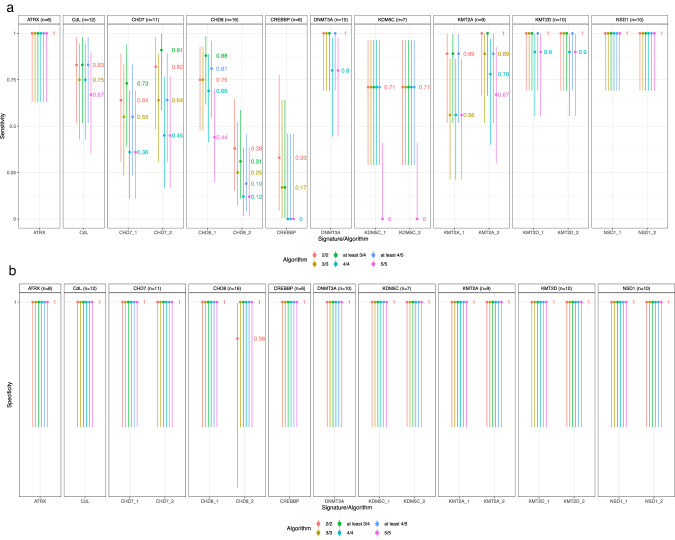
Episignature predictive performances. Panel (**a**) and (**b**) display sensitivities and specificities, respectively, according to various clustering parameters. Error bars indicate 95% confidence intervals based on a binomial distribution.

In all settings, our multiclass kNN prediction algorithm reached 100% specificity (95%CI [86–100%]), whatever the kNN parametrization, except for CHD8_2 which obtained a specificity of 96% [80–100%] under a 2/2 parametrization. As displayed on Fig. 1, five genes benefited from at least one signature reaching 100% sensitivity with multiple kNN configurations: *ATRX*, TBRS/*DNMT3A*, Kabuki*/KMT2D*, Sotos*/NSD1, WDSTS/KMT2A_1 and 2*. Robustness to kNN parametrization depended on the gene under investigation. A subset of episignatures showed good but highly variable sensitivities in the 40–90% range according to the kNN configuration used, namely CdL, CHARGE/*CHD7*, AUTS18/*CHD8*_1, and MRXCSJ/*KDM5C*_1 and 2. The sensitivity of these episignatures decreased as we increased the proportion of concordant nearest neighbours required for prediction. Two episignatures, AUTS18/*CHD8*_2 and RSTS/*CREBBP*, showed <40% sensitivities, whatever the configuration, with close to 0 sensitivity for highest numbers of concordant nearest neighbours. Overall, the “3/4” parametrization seemed to universally reach the best tradeoff given the sample size under study. Under such parametrization, inter-syndrome specificity was estimated at 100% [96%–100%] for all signatures except for CHD8_2 and both NSD1 signatures (99% [94%–100%]).

For most episignatures, sensitivity and specificity estimates from EpigenCentral, SVM predictor or non-adjusted methylation levels were similar to the results displayed in Fig. 1 (Supplementary Figs. 1–3). The main difference was observed for episignature CHD8_2, whose sensitivity rose to 100% [79.4%–100%] when using a SVM predictor or 93.8% [69.8%–99.8%] when working on non-adjusted methylation levels.

**Fig. 2:**
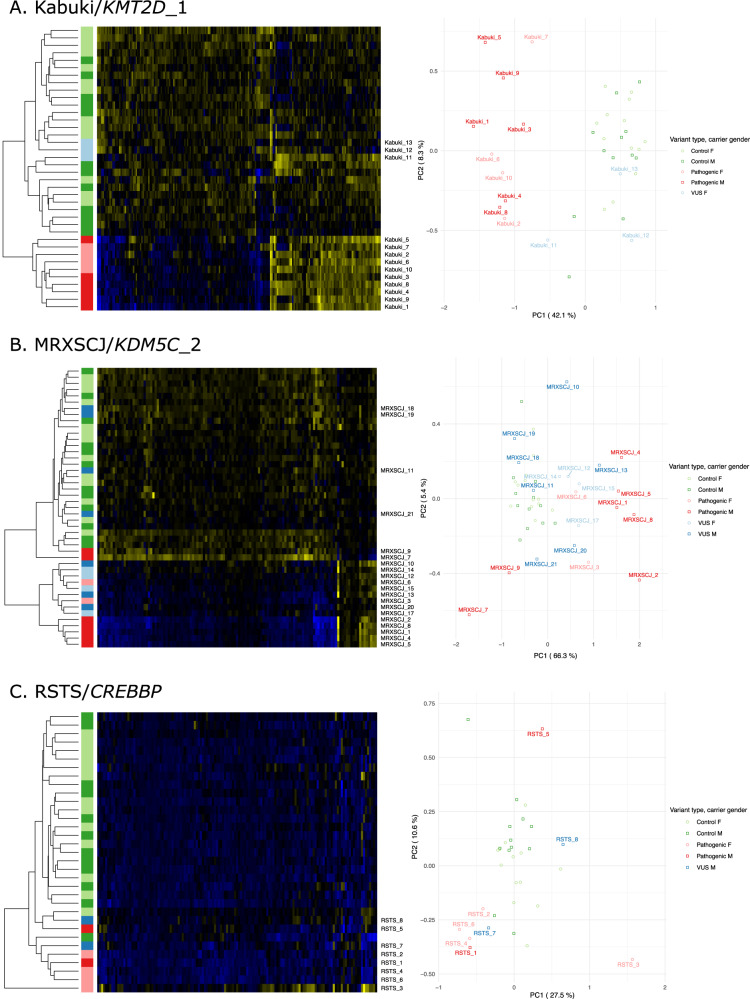
Visual inspection of three typical examples of robust, unstable and weak signatures. Respectively for (**A**) Kabuki/KMT2D, (**B**) MXRSCJ/KDM5C and (**C**) RSTS/CREBBP, each panel displays the heatmap with simultaneous hierarchical clustering of validation and testing samples on the left. Blue indicates hypo-methylated positions while yellow indicates hyper methylated positions. First principal components are represented on the right. Percentage of explained variance is added in parenthesis on each axis.

Visual inspection of PCA plots and heatmaps correlated well with sensitivity estimates. Three PCA plots and corresponding heatmaps, namely Kabuki/*KMT2D_1*, MXRSCJ/*KDM5C_2* and RSTS/*CREBBP*, chosen to best illustrate the wide spectrum of episignature performances, are presented in Fig. 2. Graphical representations for other episignatures can be viewed in Supplementary Figs. 4–13. Notably, syndromes with 100% sensitivity displayed a separation of cases and controls by a large margin, while syndromes with intermediate and unstable sensitivity suffered from a strong heterogeneity among variant carriers, with a juxtaposition of what could be called extreme and milder DNA methylation profiles. The intermediate status of a subset of profiles is clearly visible on the heatmaps. AUTS18/*CHD8*_2 and RSTS/*CREBBP* episignatures showed incomplete separation between cases and controls.

The strength of episignatures was also reflected by the percentage of methylation variance within the signature that is explained by the first axis. Indeed, the separation between cases and controls was observed on the first and major axis of variation. On an episignature like Sotos/*NSD1*, this separation accounts for 86.7% of the variance. For MRXCSJ/*KDM5C*, this number falls down to 66.5%. On the RSTS/*CREBBP* episignature, the separation between cases and controls accounts for no more than 27.5% of the variance of methylation level residuals, leaving more than two thirds of the variance to unexplained factors or noise.

Surprisingly, two carriers of *KDM5C* class 4 and 5 variants appeared as outliers on both the heatmap and first principal components (MRXSCJ_7 and MRXSCJ_9, supplementary fig. 9). Sample identity was double checked by Sanger sequencing and a global status prediction of all validation samples in search for obvious sample swaps. The first outlier is MRXSCJ_7, carrying a de novo missense variant, NM_004187.3:c.593 G > A, p.(Arg198Gln), classified as likely pathogenic. The second outlier is MRXSCJ_9, carrying a small deletion NM_004187.3:c.645_657+5del, p.? (inheritance unknown), classified as pathogenic. ACMG-AMP classification of the variants was based on a collegial decision taking in silico pathogenicity predictions, mode of inheritance, clinvar co-occurences as well as phenotype concordance into account and is not questioned.

Among the ten syndromes under investigation, six had two distinct published episignatures. Except for AUTS18/CHD8, performances were somewhat similar between the two available signatures. In contrast, our data shows that the episignatures that we called AUTS18/*CHD8*_1 [18] clearly outperformed the other one (AUTS18/*CHD8*_2) [7].

### Episignatures as a biomarker for VUS classification

Overall, we investigated 57 VUS carriers and 8 probands with a suggestive phenotype but negative exome sequencing. Sample characteristics and classification results are displayed in Supplementary Table 1. Several results deserve special attention.

Twelve VUS carriers showed methylation profiles compatible with the corresponding episignatures. From these, two samples had a discordant prediction between the two available episignatures. CHARGE_14 presented a positive episignature with the CHARGE/CHD7_1 [19] episignature but not by the CHARGE/CHD7_2 episignature [7]. The opposite was observed for the CHARGE_12 sample. Both segregated properly with cases in the PCA plot made from the CHARGE/CHD7_2 probes but separation between cases and controls was milder for this episignature, which can cause classification errors (Supplementary Fig. 7).

In fact, several episignatures, even among those with perfect separation and 100% sensitivity, found themselves challenged in practice by VUS with intermediate methylation profiles. Such a phenomenon was observed for sample Kabuki_11, which as a result presented a conflicted positive Kabuki/KMT2D_2 episignature but negative for Kabuki/KMT2D_1 (Fig. 2A). Although such profiles were expected and observed for female carriers of X-linked syndromes, two male *KDM5C* VUS carriers also presented intermediate profiles (Fig. 2B). Of course, this situation might arise from unobserved heterogeneity among cases, and such undecisive scenarios could be settled by the analysis of a larger dataset.

One proband carrying a pathogenic *CHD7* complete duplication was included among VUS. This duplication presented a negative episignature, but PCA plot inspection revealed that it was projected on the opposite end of the first axis. This observation is consistent with the haploinsufficiency mediated pathogenicity in CHARGE syndrome [31].

Among patients with negative episignatures, Sotos_17 was later discovered to harbour a pathogenic variant in *PTCH1*, thus confirming its differential diagnosis.

Among clinical hypotheses, only one proband with a Sotos syndrome clinical phenotype harboured positive Sotos/NSD1 episignatures, suggesting that the patient actually presents this disorder and that further genetic analyses should be proposed to identify the causal variant.

## Discussion

To our knowledge, we provide the first independent and unbiased evaluation of 16 episignatures spanning 10 neurodevelopmental disorders, in terms of predictive accuracy and robustness. All data were newly generated for this project, and none were included in previous training sets used for the selection of probes and detection of episignatures. For every signature, the multiclass and stringent kNN strategy guaranteed perfect specificity, with regard to both normal controls and other syndromes. This specificity estimation is based on 25 unaffected controls, but all matched to cases from a technical point of view. Resorting to large control sets from the GEO platform could have increased sample size but spurious differences between datasets would have biased our estimations. The perfect inter-syndrome specificity brings confidence to the interpretation of probands with a suggestive phenotype but with negative exome sequencing. On the other hand, sensitivity was highly heterogeneous among syndromes, with close results between kNN, SVM and EpigenCentral predictors. Combined with sensitivity estimates, visual inspection of PCA plots and heatmaps revealed that, essentially, episignatures could be split into three groups: (i) robust signatures ready for confident use in a diagnostic setting (ii) signatures of reasonable but unstable predictive abilities, facing challenges in practice (iii) weak signatures that are not ready for use in a diagnostic setting. The proportions of probes with large methylation gap between cases and controls seem highly evocative of this partition.

The first group includes *ATRX*, Sotos/NSD1, TBRS/*DNMT3A* and Kabuki/*KMT2D* robust signatures. Pathogenic variants in these four genes led to a perfect separation between cases and controls, displaying a robust 100% sensitivity. This observation is well documented in the literature, namely that Sotos and TBRS syndromes are known to cause a drastic hypo-methylated signature among carriers with large overlap between differentially methylated probe sets [7, 11, 20, 21, 26]. The similarities between signatures caused a slight risk of misclassification, even within our multiclass prediction framework. Increasing sample size should help decipher the biological meaning of these overlaps as well as reduce misclassifications. Inversely, the overlap between Kabuki/KMT2D_1 and Kabuki/KMT2D_2 is marginal (37 out of 153 and 221 probes respectively), reminding us that two distinct signatures can be equally powerful thanks to the highly correlated structure of CpG methylation levels. Besides, despite these perfect performances on the validation set, some VUS remained impossible to classify in practice within *ATRX* and *KMT2D*, raising the question of whether similar issues could arise in practice within *NSD1* and *DNMT3A* genes, should we increase the number of VUS under investigation.

The second group consist of CdL, CHARGE/*CHD7*, AUTS18/*CHD8_1*, MRXCSJ/*KDM5C*, *WDSTS/KMT2A*, episignatures. These signatures showed reasonable sensitivity, making them effective biomarkers for VUS classification in theory, but intermediate profiles rendered sensitivity estimates dependent on classifier parametrization and interpretation complex in practice. Co-occurences of milder episignatures with milder phenotypes have been previously reported [16, 34–36]. Here, AUTS18_2 was the father of AUTS18_1, both carrying the same *CHD8* pathogenic variant. While the daugther showed typical features of AUTS18/CHD8 (intellectual disability, autism spectrum disorder and macrocephaly), her father only displayed macrocephaly without any known neurodevelopmental features. Nevertheless, his methylation profile was without any doubt positive. That single observation does not support the hypothesis that milder phenotype inevitably coincides with intermediate profiles, at least regarding AUTS18/*CHD8*. We also identified two samples with opposite methylation patterns in *KDM5C*. Close attention revealed that the two variants affected the same exon. Works by Ugur et al. showed that amino acids 199 to 218 of KDM5C protein—in which these two variants are located—define a very specific functional domain, suggesting a potential domain-specific effect [32]. It is impossible to draw a firm conclusion on two occurences, but it is conceivable that pathogenic variants in this precise region should induce a distinct impact on DNA methylation. This scenario has already been described for *ADNP* which has a main episignature and a second one restricted to a specific protein domain [33]. More samples are required to confirm this hypothesis.

On the lower end of the spectrum are RSTS/*CREBBP* and AUTS18/CHD8_2 signatures. Our data advise against the use of these episignatures in a diagnostic setting. Reasons may be either biological or technical. Perhaps the methylation impact is not strong enough or cases suffer from a diversity which has not been understood yet. The low rate of positions reaching more than a 5% absolute methylation gap suggest these signatures suffer from winner’s curse and overfitting to a small discovery set (5 cases for AUTS18/CHD8_2). More recently, the same team reported in [11] an increased sample size of 28 *CHD8* cases but no update was published regarding the probe list. In the same publication, eight new episignatures have been similarly trained on <5 samples. The SVM predictor improved the sensitivity of CHD8_2 by reinforcing the weight of the few CpG positions that remain differentially methylated on the validation dataset while discarding non-reproducible ones. This apparent increase in sensitivity is probably at the cost of some new overfitting.

A recent review on episignature provided several illustrations of the complexity of interpreting episignatures in practice, be it related to intermediate profiles with *SMARCA2* or *HNRNP* examples or the existence of gene regions that evade the signature for *EZH2* and *SRCAP* genes [34]. Our work suggest that these scenarios are much more common than could be expected. Without questioning the validity of corresponding episignatures, intermediate profiles and local exceptions demonstrate that episignatures cannot be used as automated binary tools. It is important that predictions should be challenged by careful visual and expert inspection. Precise interpretation of these complex episignatures requires further investigation of more samples to (i) confirm and understand the implications of diverse methylation profiles and (ii) investigate the existence of regions that may escape the signatures. A larger sample size would allow for genotype/methylation and phenotype/methylation correlation analyses to provide informed and accurate genetic counselling to carriers. As always in the molecular biology diagnostic process, biological and clinical expertise about the neurodevelopmental syndrome is mandatory to make an analysis reliable.

From a technical point of view, a limitation of our study is that all probes in the signature contributed equally to the prediction, contrary to other machine-learning models that combine probes with more flexibility. However, with only about ten samples per syndrome, kNN provided a more reasonable and robust approach which limits the introduction of a new level of overfitting. With larger sample sizes, other methodological options could be devised.

### Supplementary information

Supplementary figures

Supplementary tables

R script

## Data Availability

The datasets generated during and/or analysed during the current study are available from the corresponding author on reasonable request. Probe lists and average residual methylation levels per probe and syndrome are given in Supplementary Tables 3 and 4, respectively.

## Code availability

Major code steps permitting to reproduce the analyses are given in Supplementary Information.
