# Supplementary material for: Episignatures in practice: independent evaluation of published episignatures for the molecular diagnostics of ten neurodevelopmental disorders
Source: Eur J Hum Genet. 2023 Oct 23;32(2):190–9. doi: 10.1038/s41431-023-01474-x (PMC10853222; doi:10.1038/s41431-023-01474-x)

Supp Figure 1. Sensivity and specificity of kNN compared to Epigencentral, with Illumina or FunNorm normalizations.

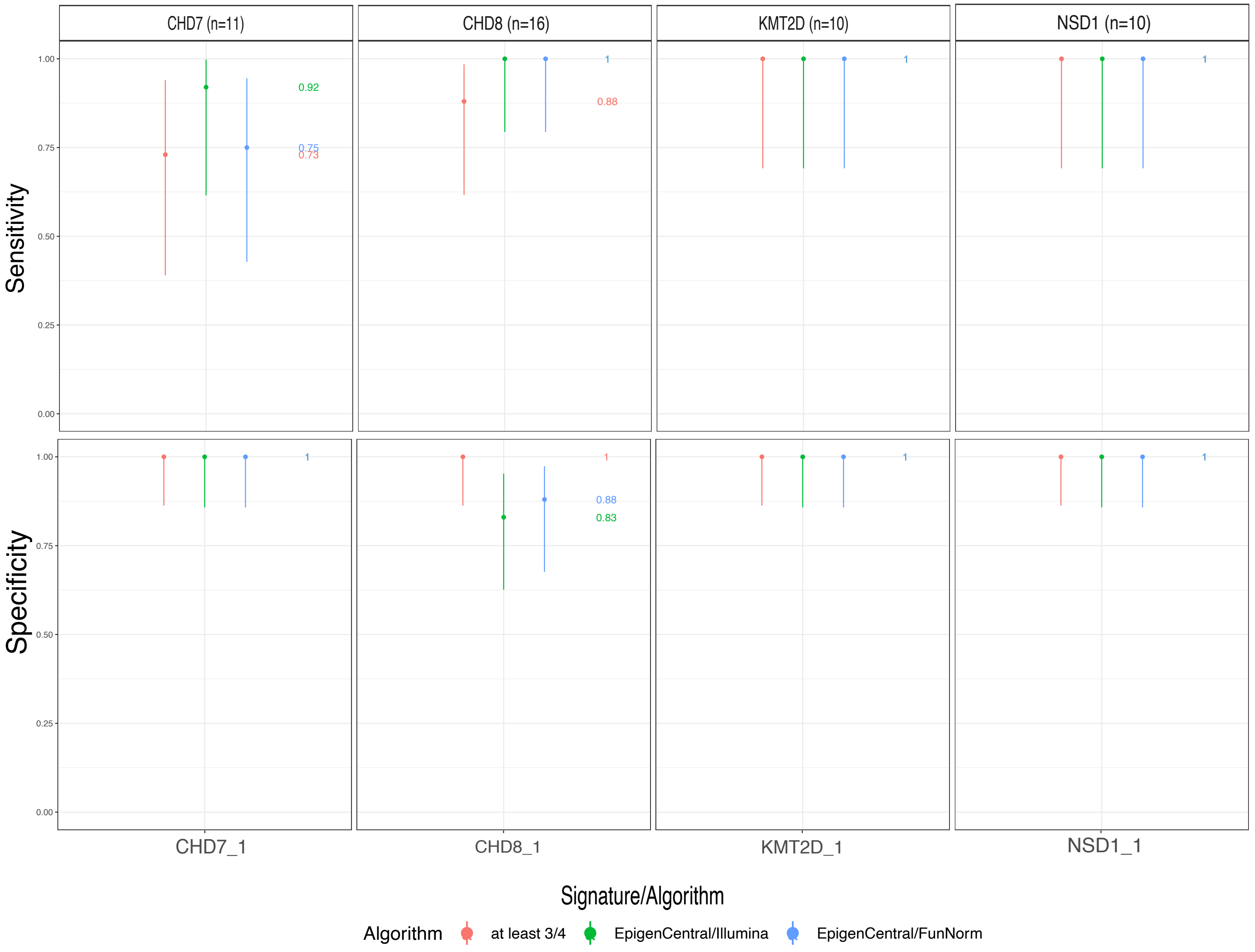

Supp Figure 2. Sensivity and specificity of kNN compared to svm,

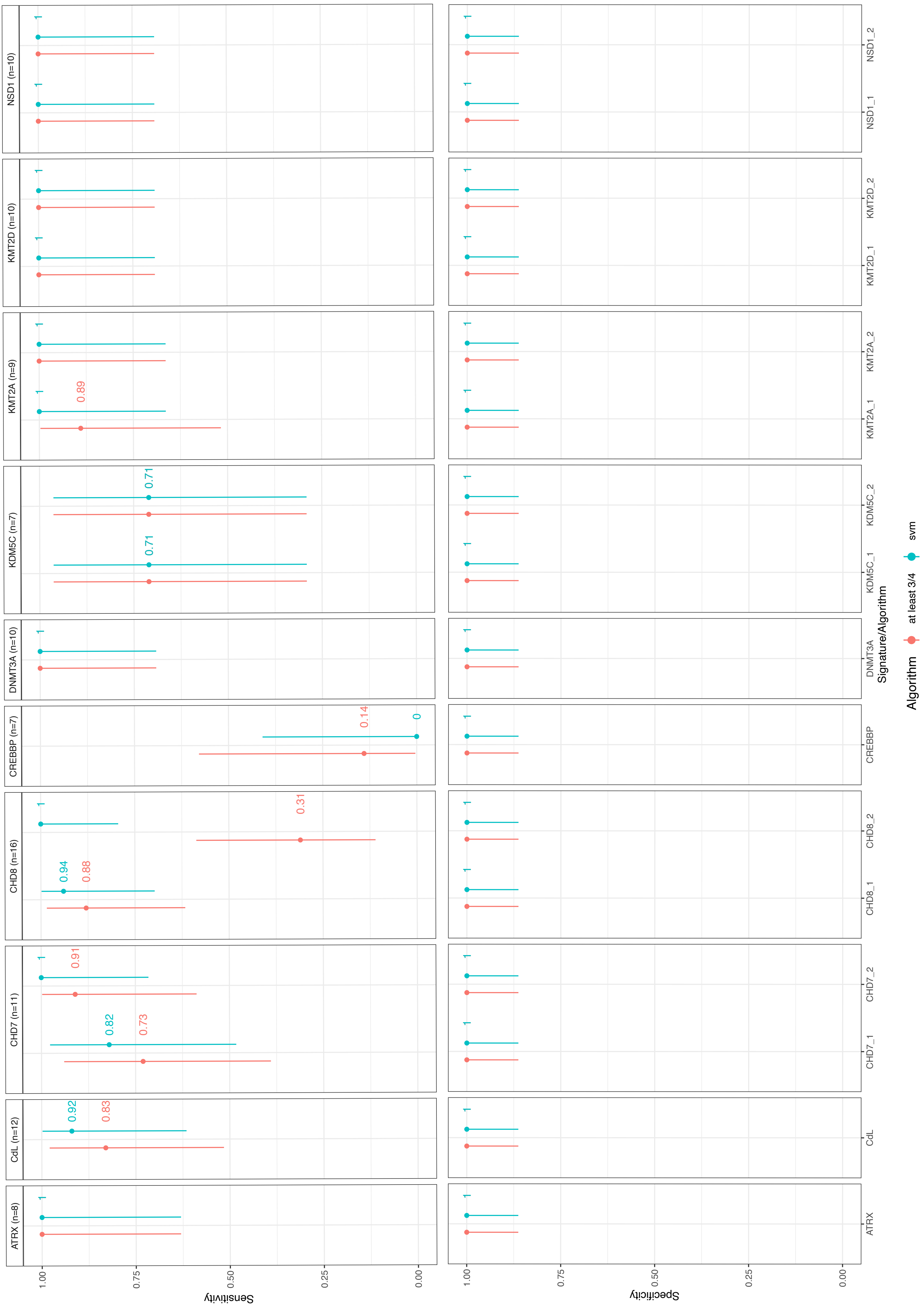

Supp Figure 3. Sensivity and specificity without adjustment for confounders

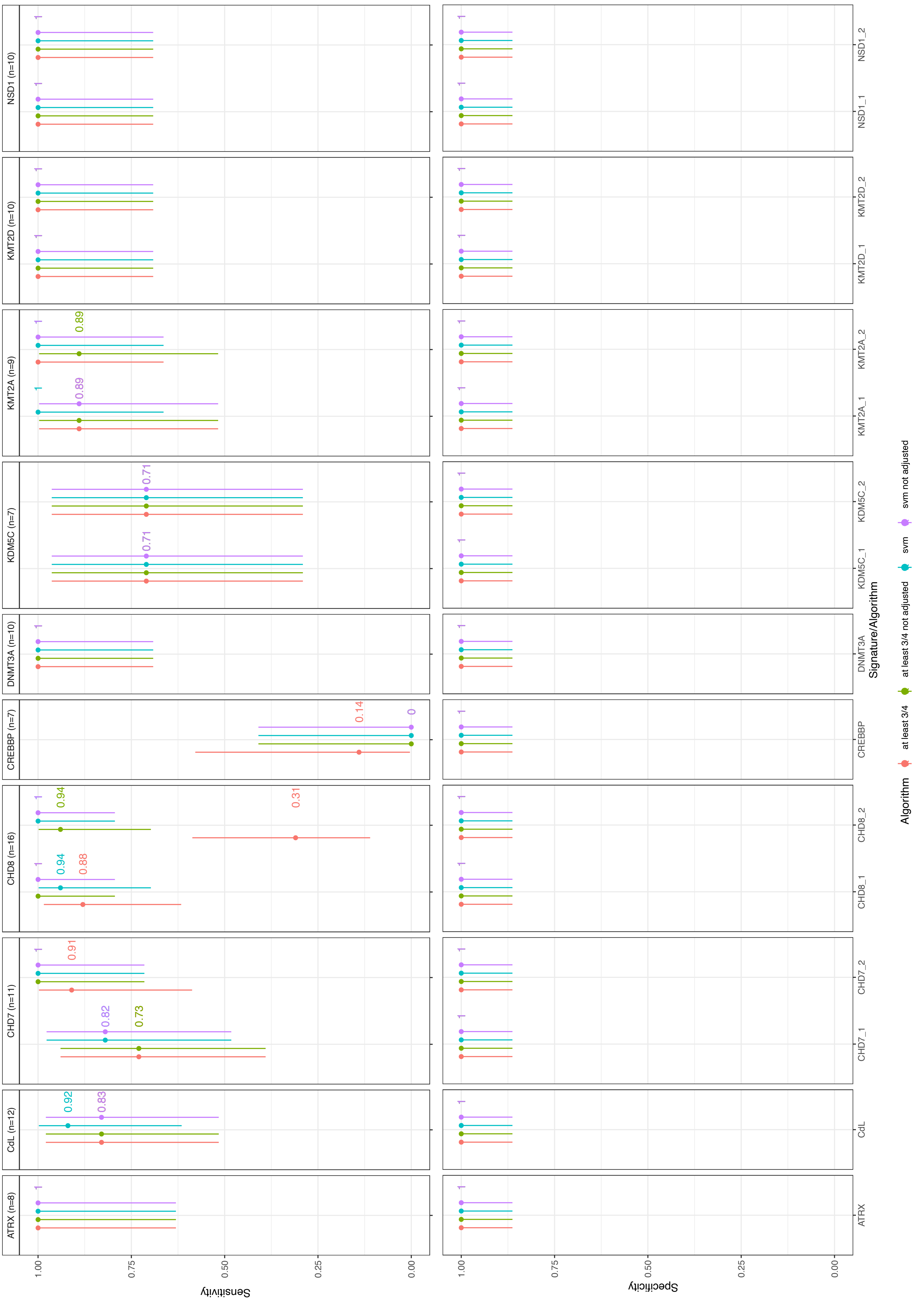

Supp Figure 4. ATRX signature, visual inspection

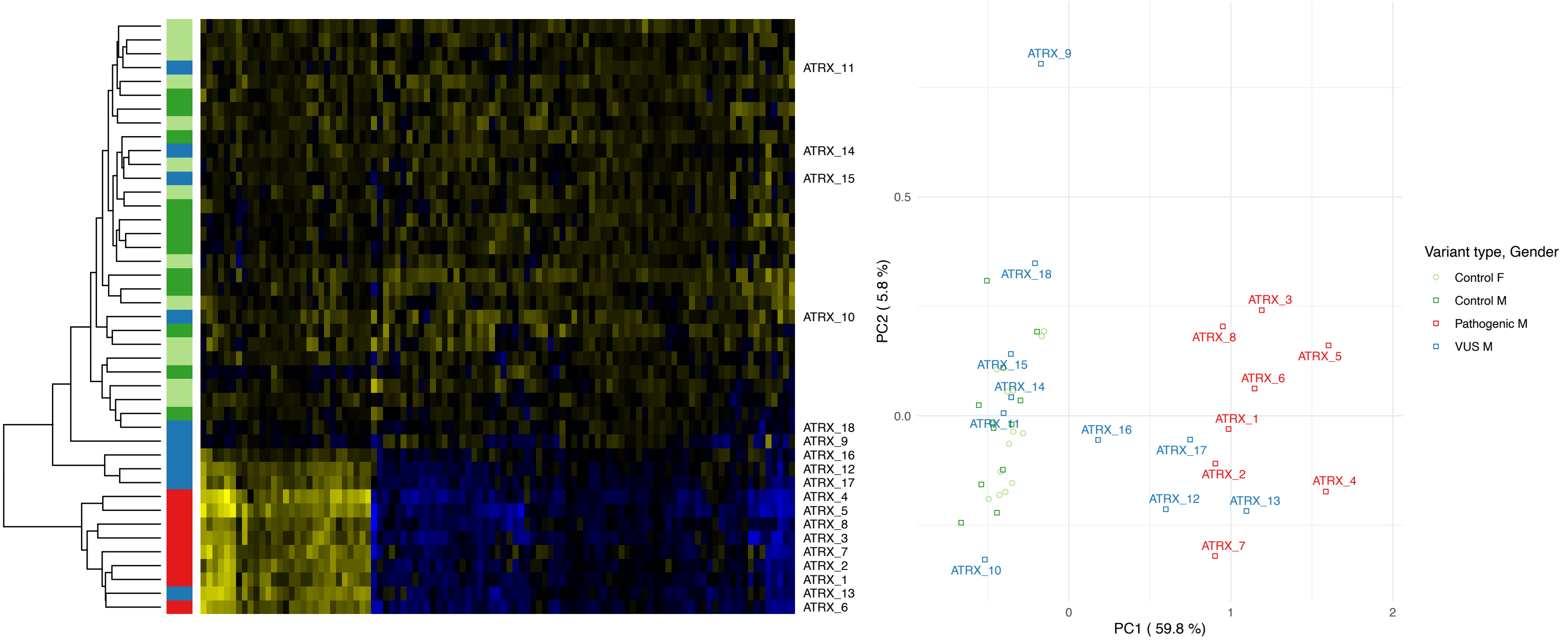

Supp Figure 5. CdL signature, visual inspection

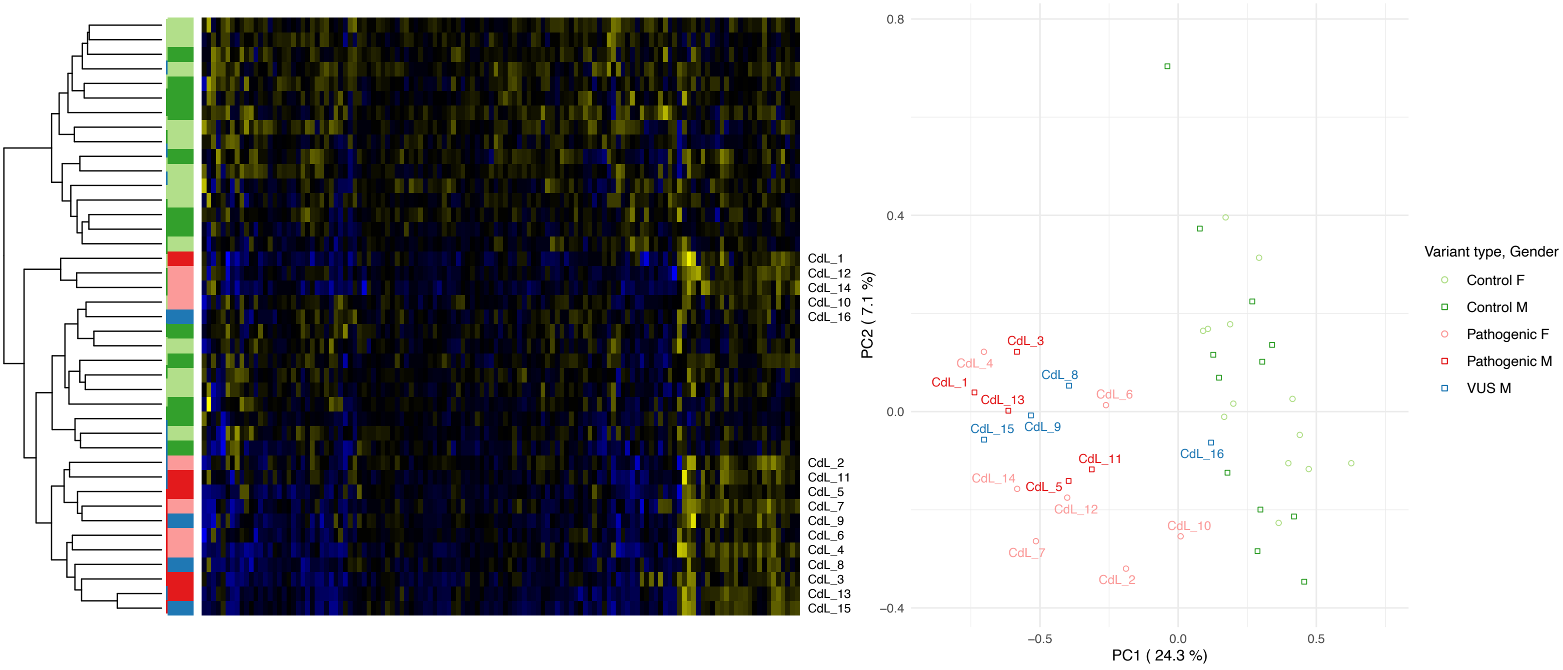

Supp Figure 6. CHARGE/CHD7 signatures, visual inspection

A. CHARGE/CHD7_1

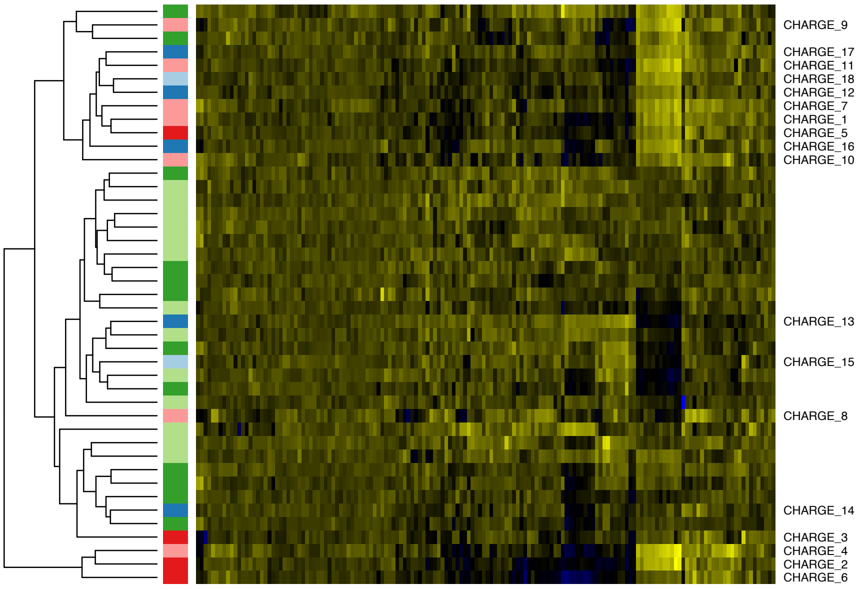

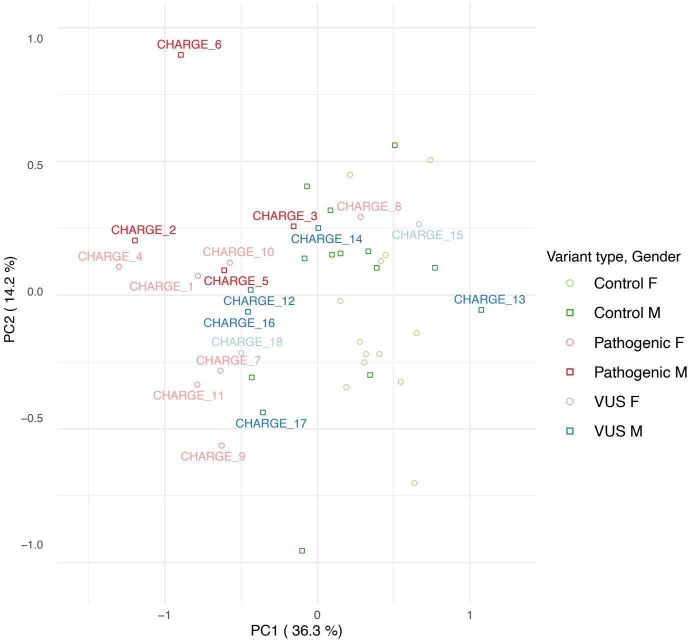

B. CHARGE/CHD7_2

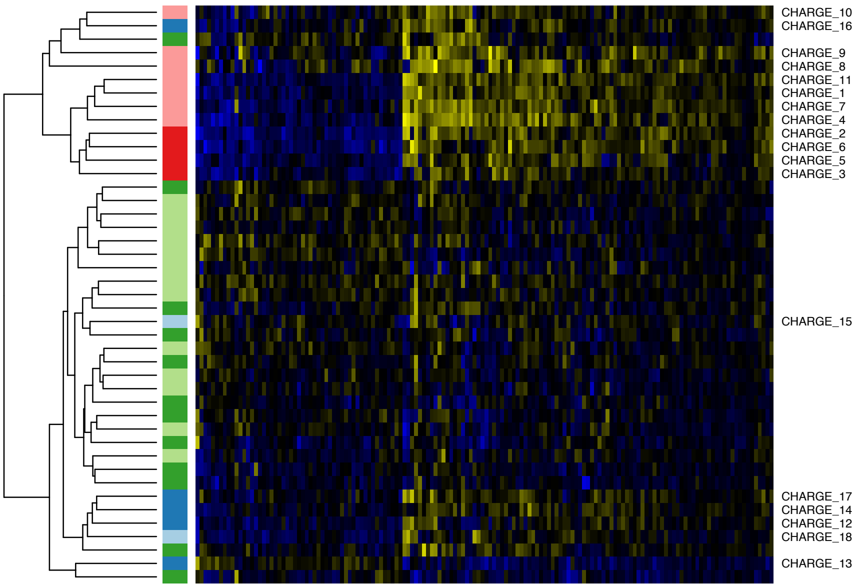

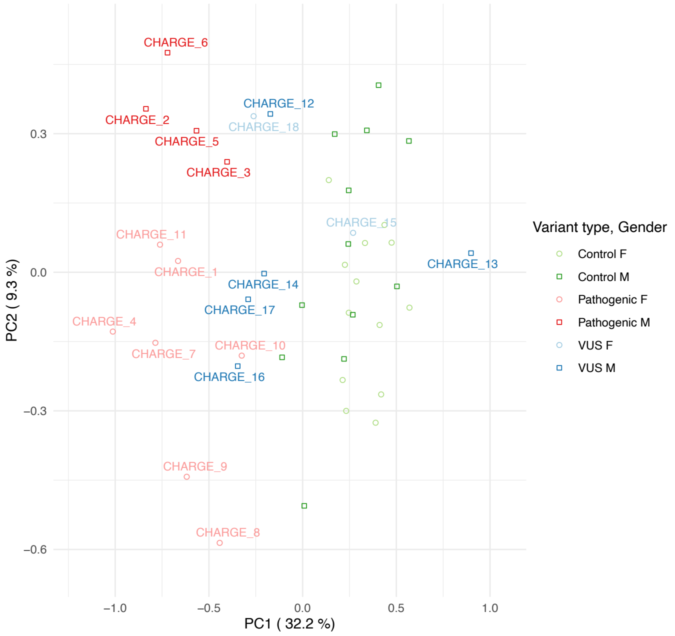

Supp Figure 7. AUTS19/CHD8 signatures, visual inspection

A. AUTS18/CHD8_1

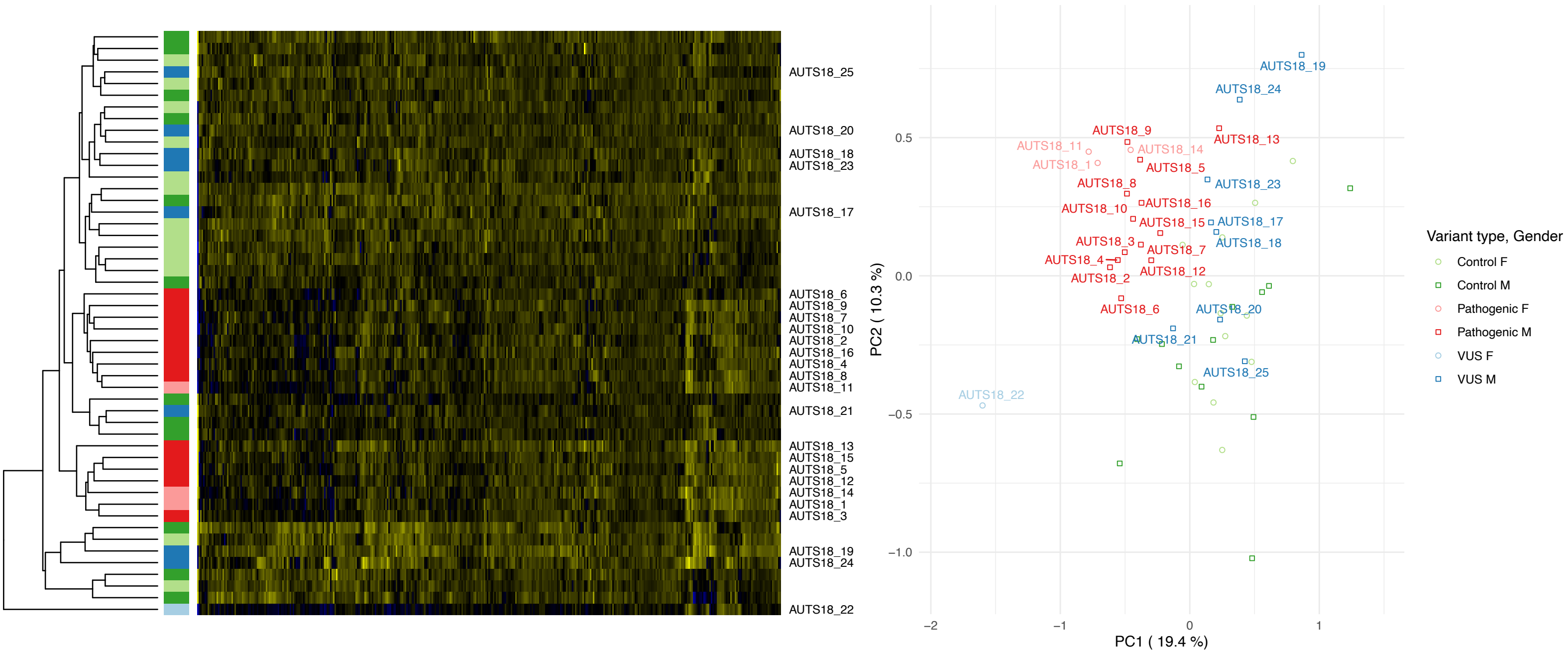

B. AUTS18/CHD8_2

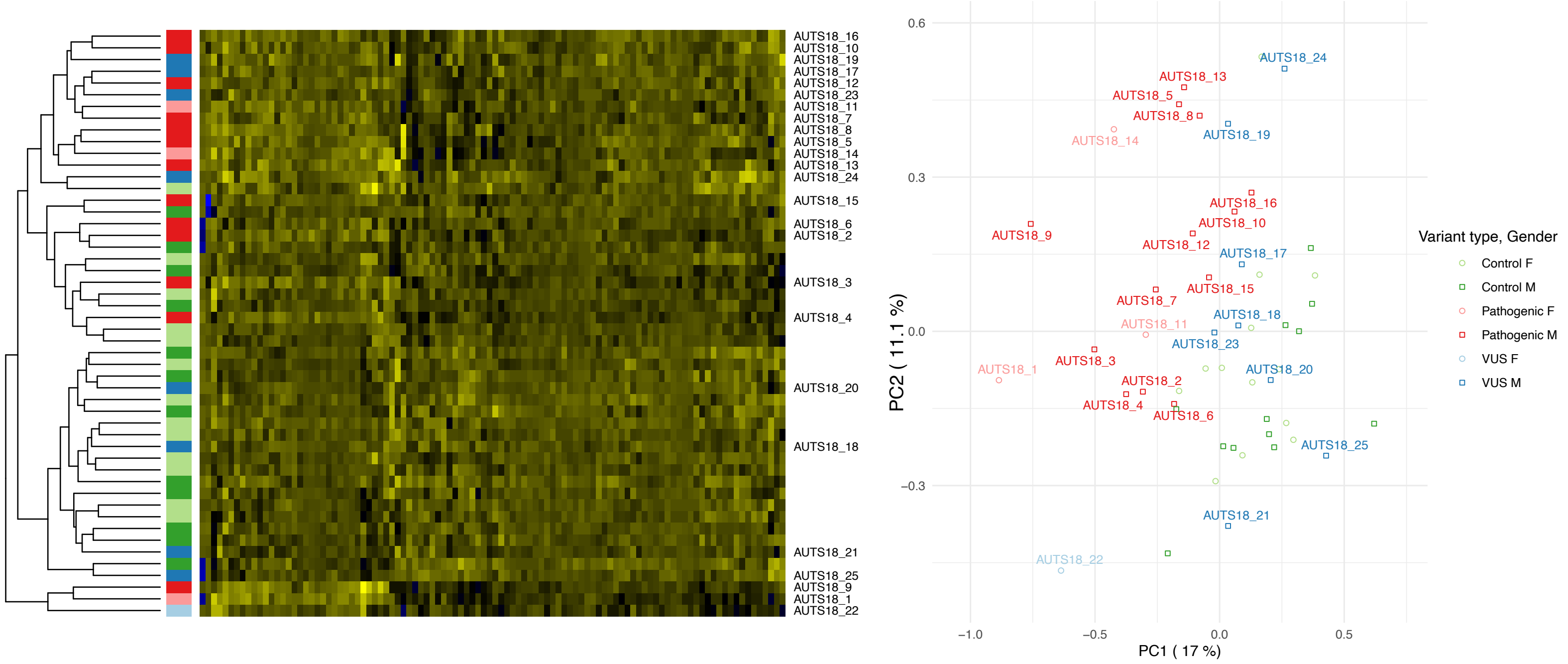

Supp Figure 8. RSTS/CREBBP signature, visual inspection

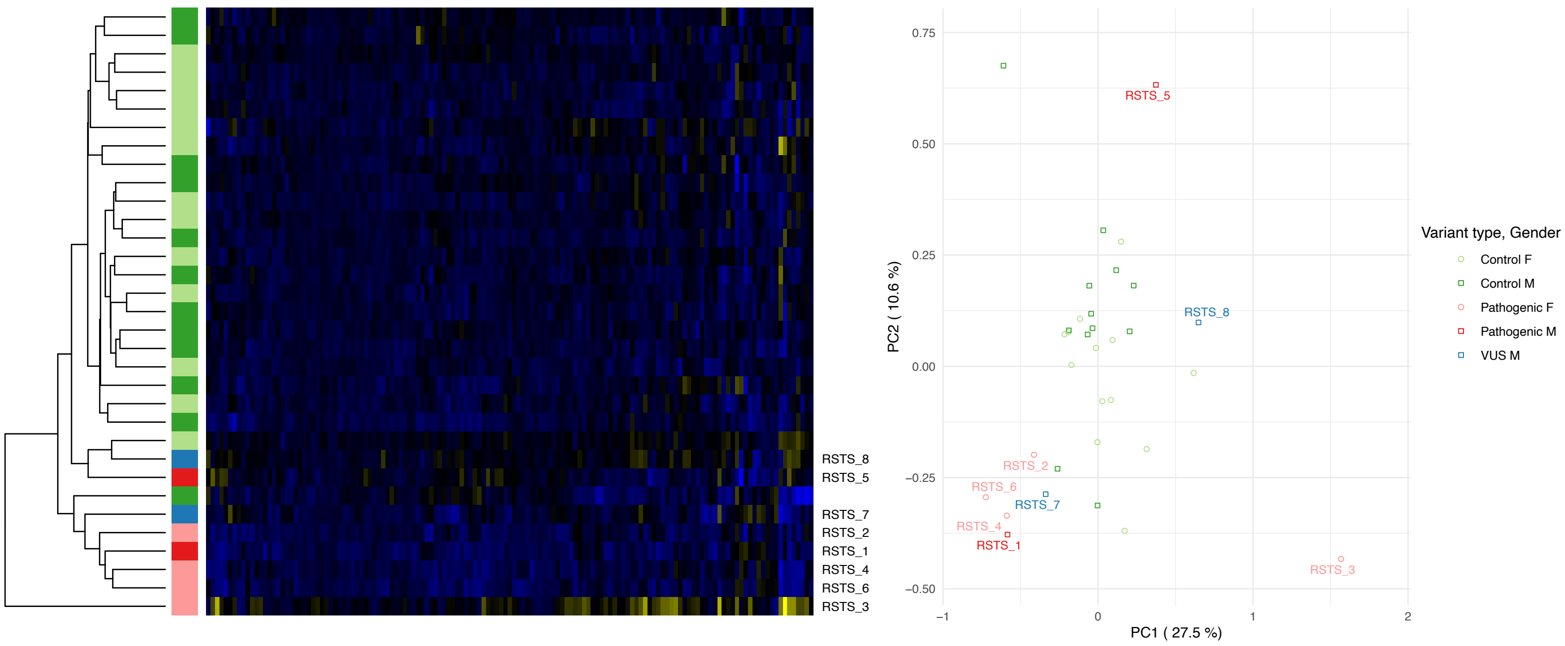

Supp Figure 9. TBRS/DNMT3A signature, visual inspection

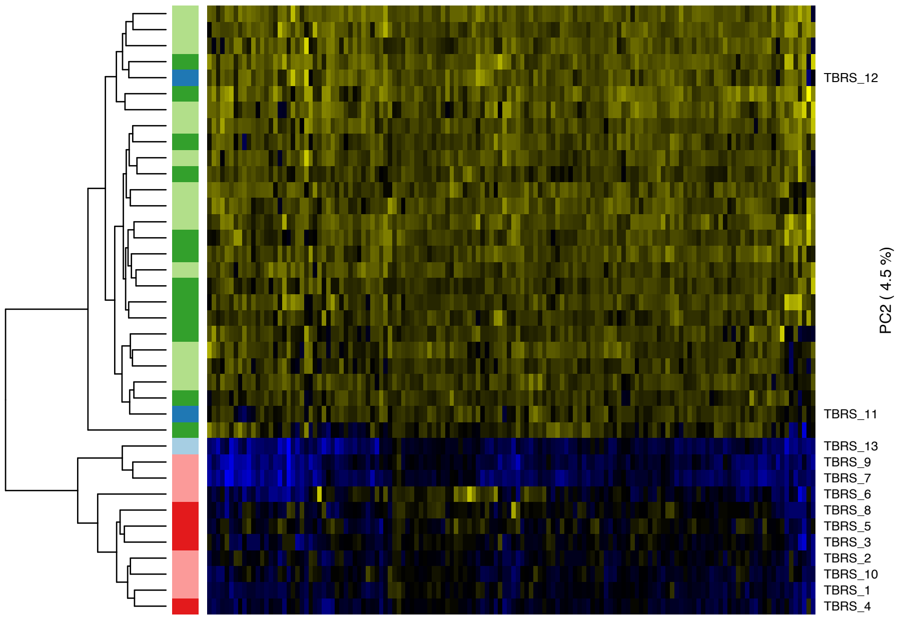

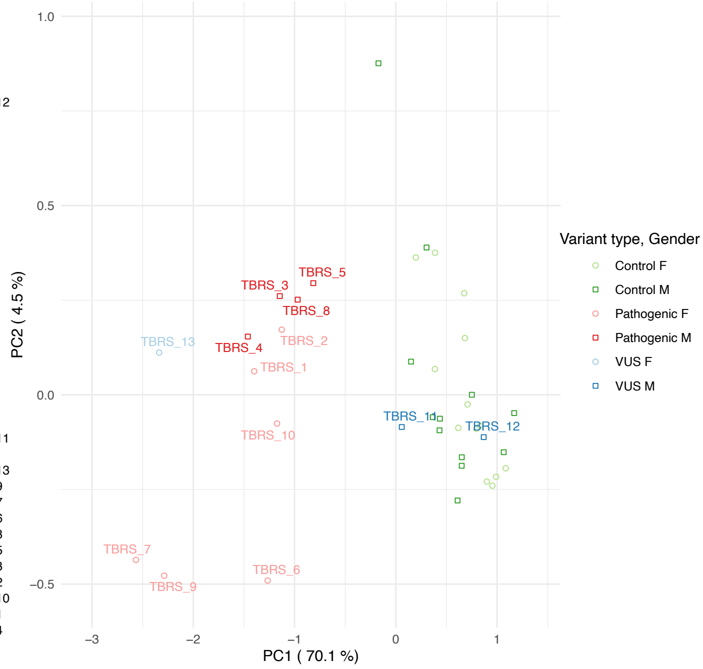

Supp Figure 10. MRXSCJ/KDM5C signatures, visual inspection

A. MRXSCJ/KDM5C_1

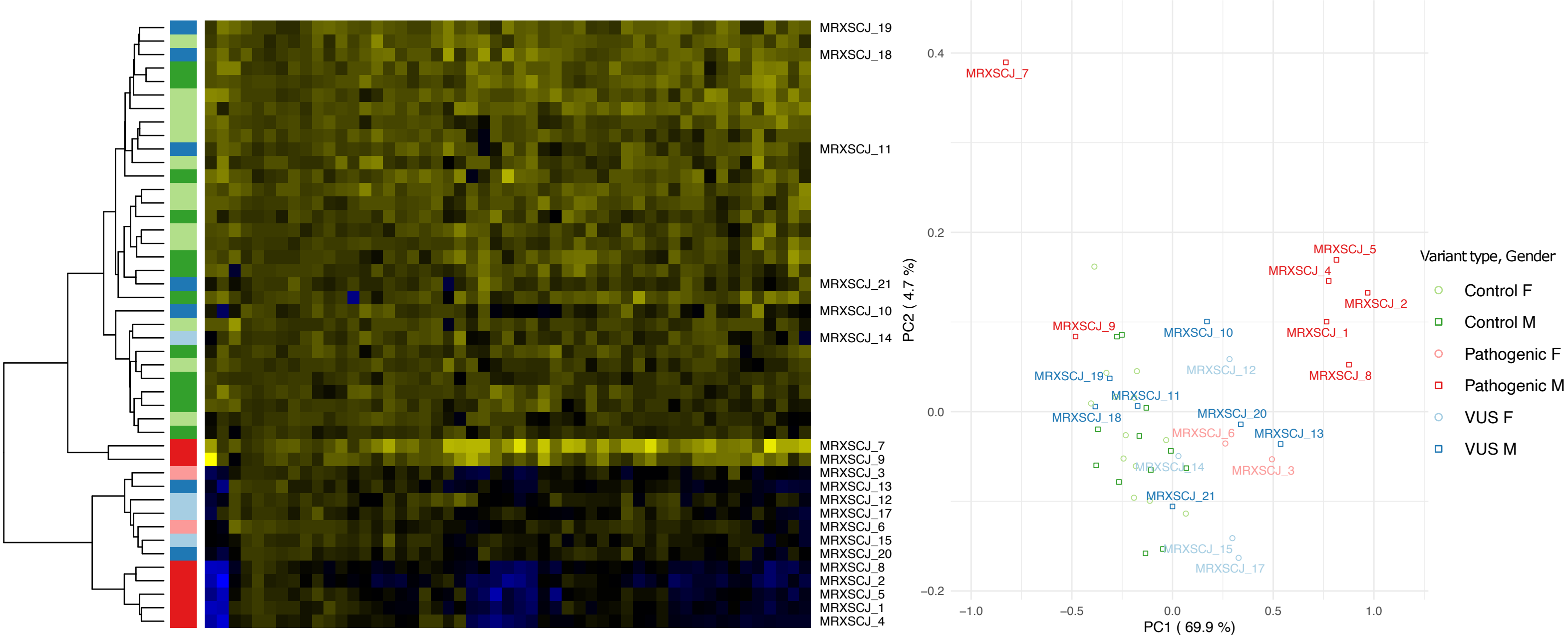

B. MRXSCJ/KDM5C_2

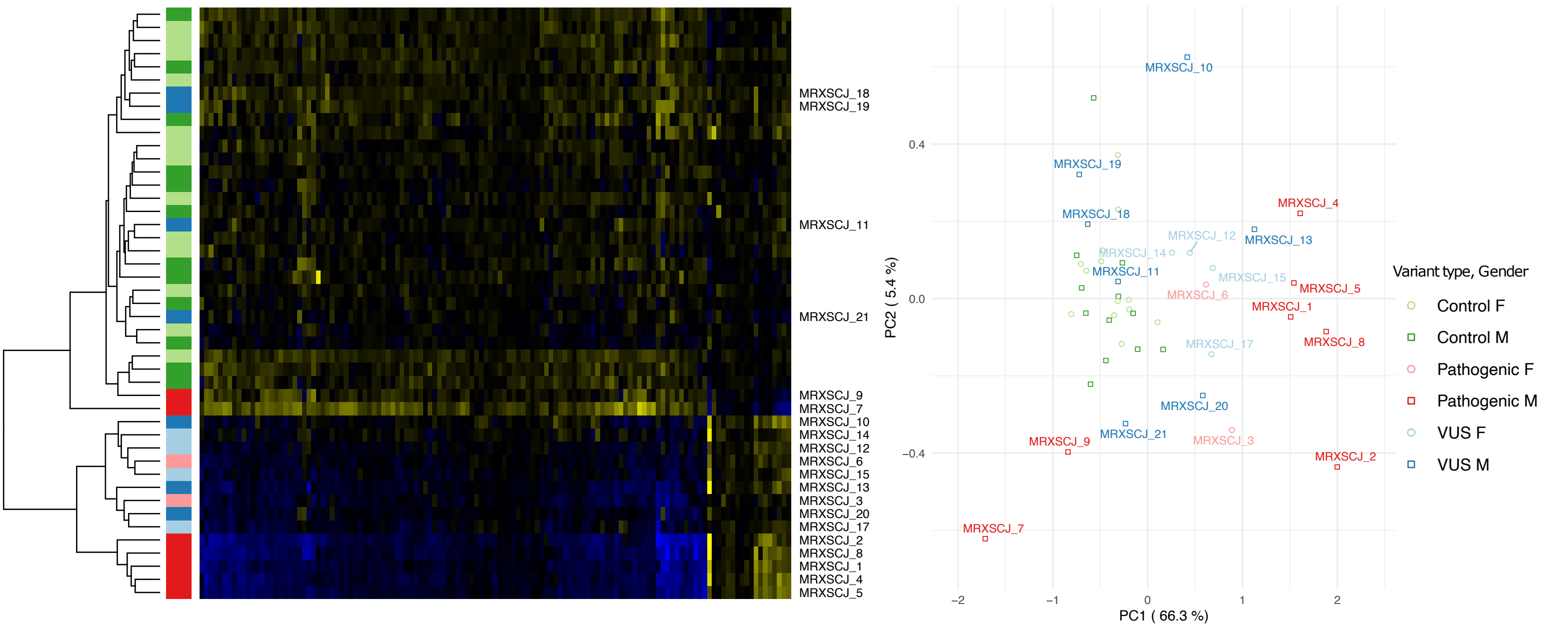

Supp Figure 11. WDSTS/KMT2A signatures, visual inspection

A. WDSTS/KMT2A_1

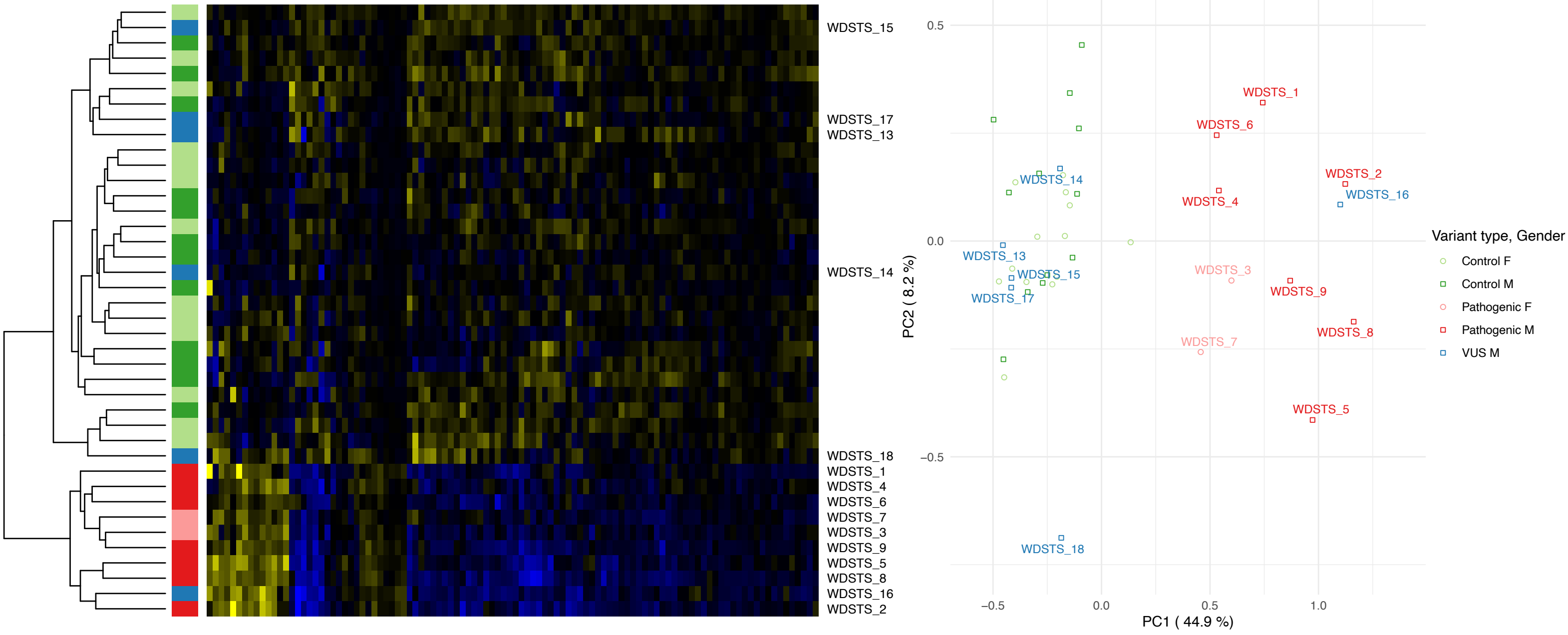

B. WDSTS/KMT2A_2

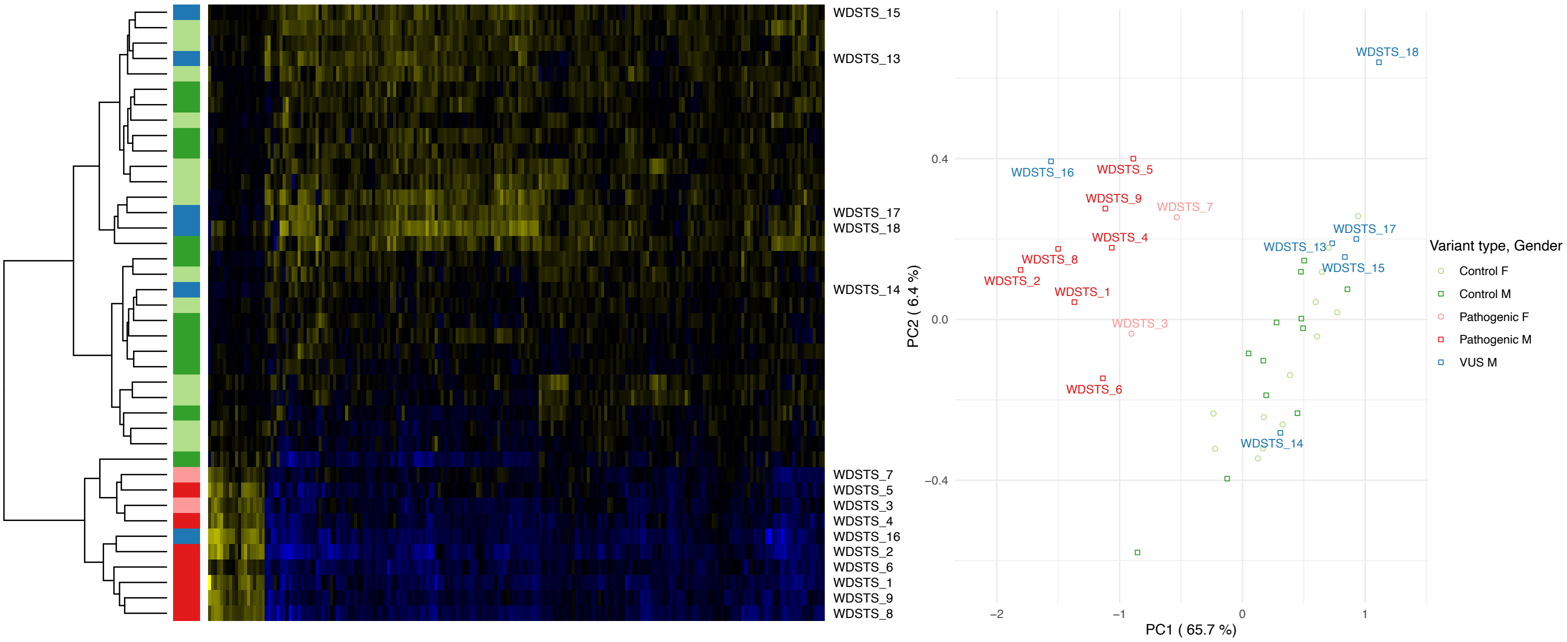

Supp Figure 12. Kabuki/KMT2D signatures, visual inspection

A. Kabuki/KMT2D_1

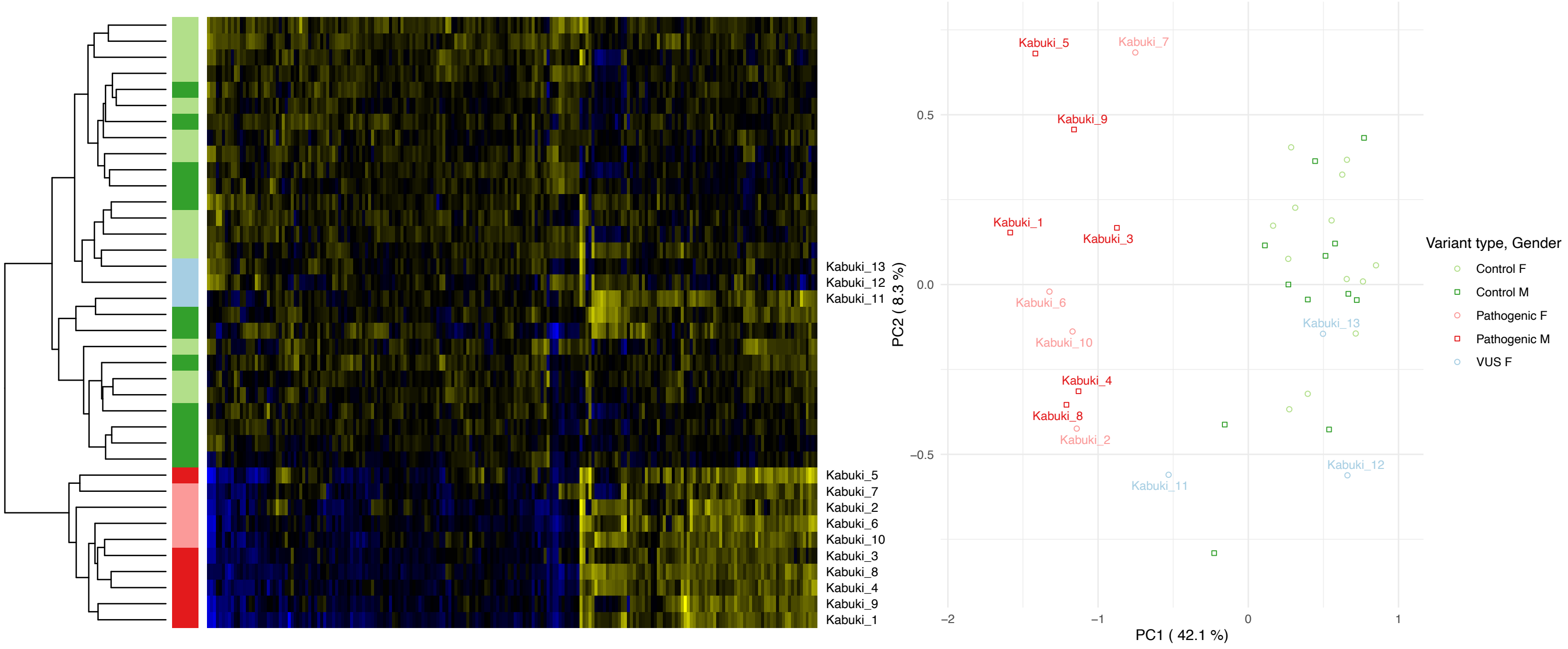

B. Kabuki/KMT2D_2

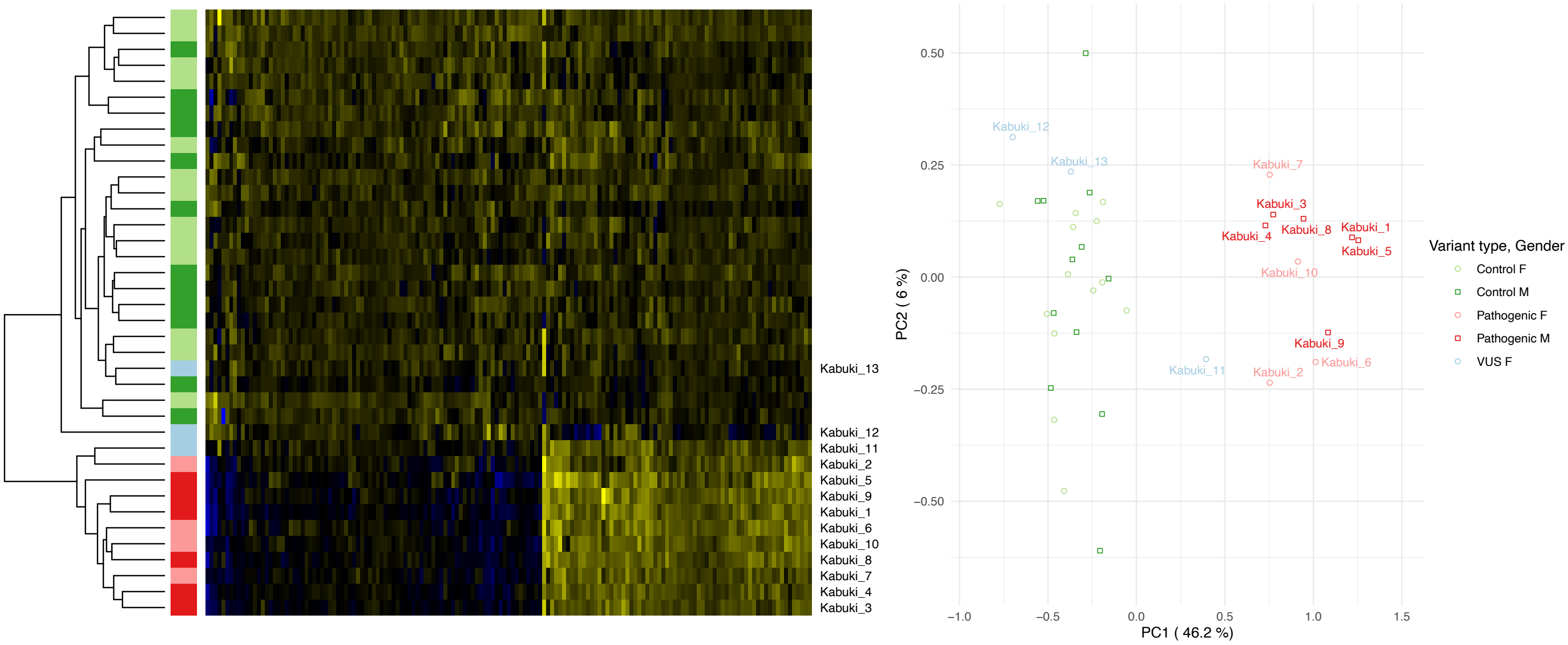

Supp Figure 13. Sotos/NSD1 signatures, visual inspection

A. Sotos/NSD1_1

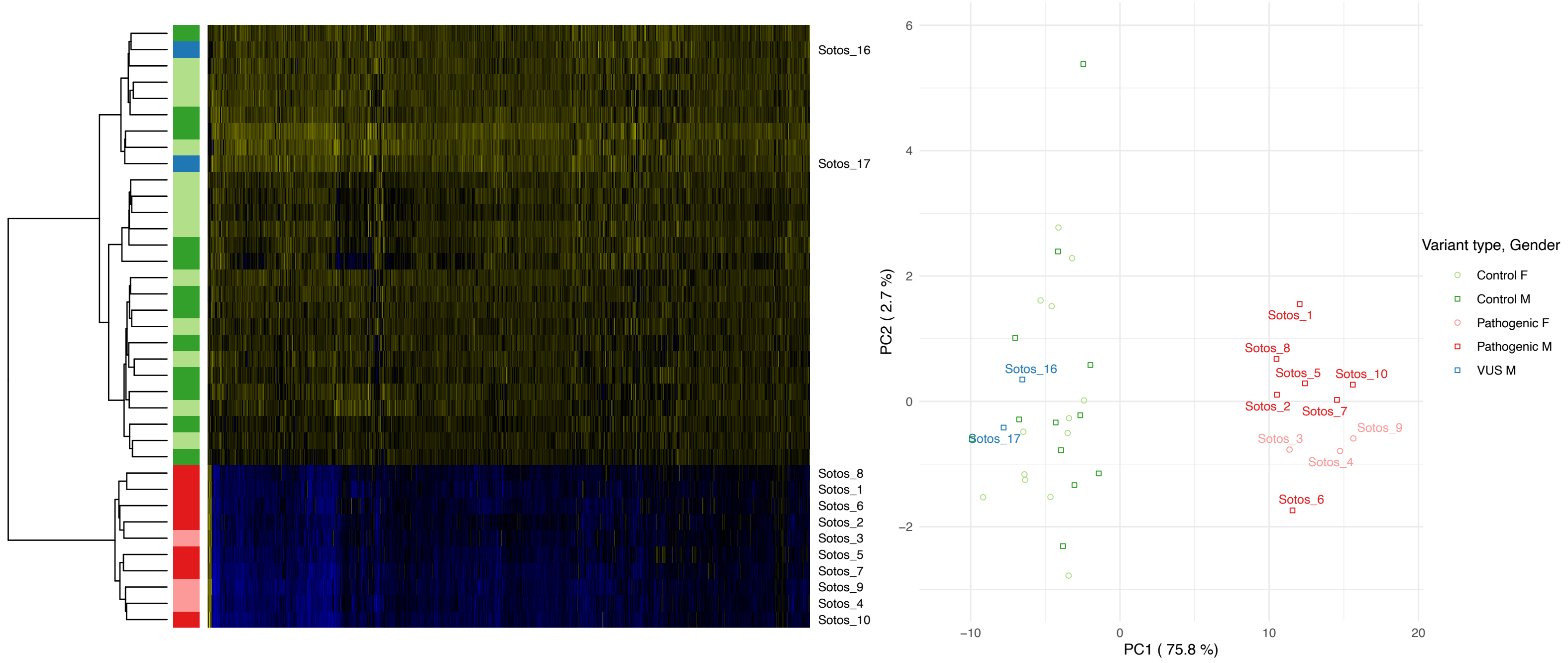

B. Sotos/NSD1_2

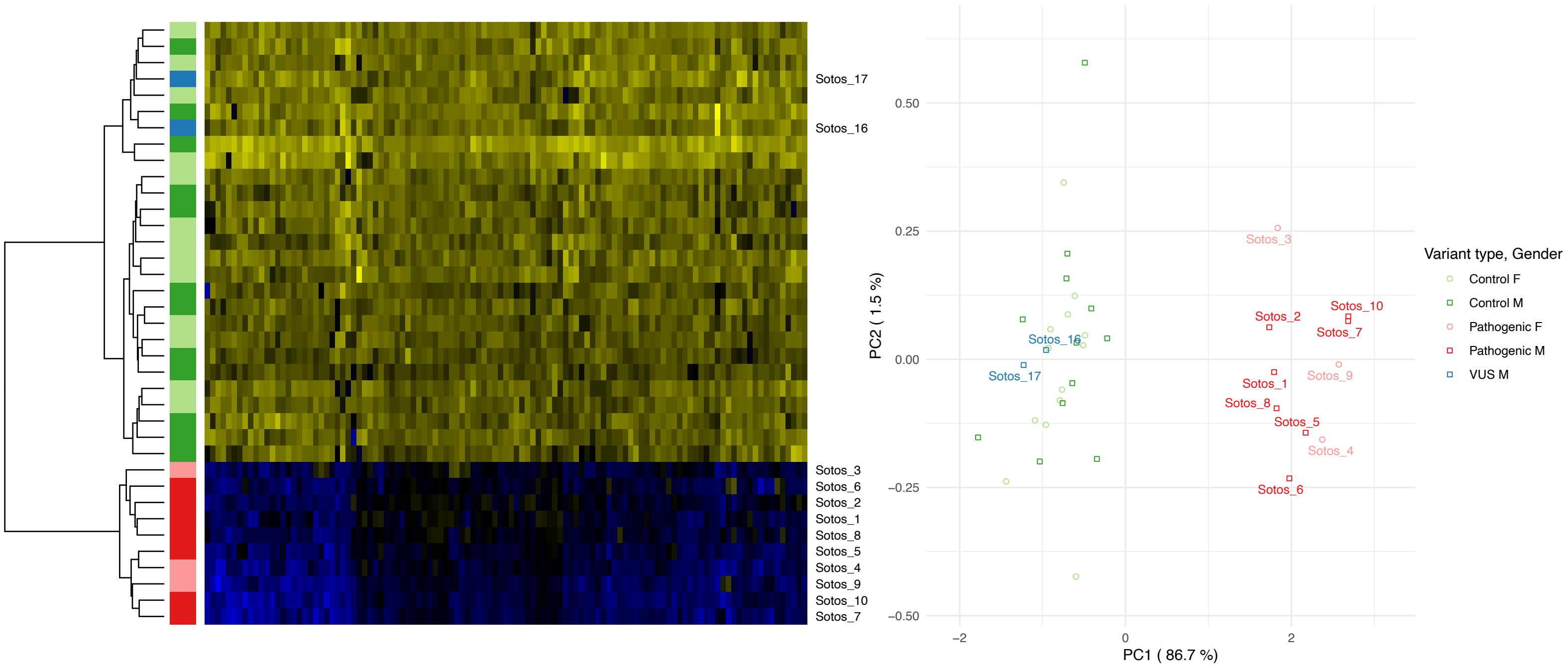

Supplement: Supplementary file 1 — Supplementary figures [file 41431_2023_1474_MOESM1_ESM.pdf]
